# A self-organizing developmental framework for the head direction ring attractor

**DOI:** 10.1007/s10827-026-00955-4

**Published:** 2026-10-17

**Authors:** Shujia Liu, Bailu Si, J. Michael Herrmann

**Affiliations:** 1 School of Systems Science, Beijing Normal University, Beijing, China; 2 School of Informatics, University of Edinburgh, Edinburgh, United Kingdom; 3 Chinese Institute for Brain Research, Beijing, 102206 China

**Keywords:** Head direction system, Ring attractor, Self-organization, Travelling waves, Structural plasticity, Visual calibration

## Abstract

The head direction (HD) system provides a continuous representation of the animal’s facing direction and is a core component of the spatial navigation network. Although mature HD circuits have been studied extensively, their developmental origin remains unclear, particularly because young animals already show attractor-like internal coherence before stable visual anchoring is established. Here we propose a developmental computational framework of the lateral mammillary nucleus and dorsal tegmental nucleus (LMN–DTN) circuit, in which a functional HD ring attractor emerges through staged self-organization without preconfigured topology or early external supervision. In the model, a randomly connected LMN network first generates stable travelling waves through relative refractoriness. These waves provide a temporal scaffold for subsequent learning. Local STDP-like and structural plasticity then consolidate wave-based statistics into locally enhanced recurrent connectivity, followed by direction-selective shift-feedback learning in the DTN*→ *LMN pathway. At a later stage, recruited long-range inhibition transforms the travelling-wave manifold into a stable single activity profile (bump) attractor. Visual input acts mainly as a late calibration signal for global integration gain: it improves short-timescale path integration, but does not remove long-term drift. The framework provides a testable account of the developmental dissociation between early internal organization and later sensory anchoring. More broadly, it suggests that key components of classical ring attractor models, including local recurrent structure, direction-selective shift feedback, and late stabilizing mechanisms, can arise through a staged developmental process rather than relying on a preconfigured network architecture.

## Introduction

The head direction (HD) system is a core component of the spatial navigation network (McNaughton et al., 2006). It continuously represents the animal’s facing direction and maintains this internal representation through the combined influence of self-motion and external sensory cues (Taube et al., 1990; Taube, 2007; Angelaki & Laurens, 2020). Although the mature HD system has been studied extensively (Sharp et al., 2001a; Clark & Taube, 2012; Cullen & Taube, 2017; Angelaki & Laurens, 2020; Taube, 2025), its developmental origin remains unclear (Tan et al., 2015; Bjerknes et al., 2015; Bassett et al., 2018).

Developmental experiments show that before eye opening, when stable visual anchoring has not yet been established, the rat HD system already exhibits substantial internal organization (Tan et al., 2015; Bassett et al., 2018). In downstream regions such as the anterior dorsal thalamic nucleus (ADN) and postsubiculum / dorsal presubiculum (PoS / PrSd), HD activity can be recorded before eye opening, and relative coupling within the population already shows attractor-like coherence (Bjerknes et al., 2015; Bassett et al., 2018). At the same time, absolute directional tuning remains unstable and is prone to drift or reset (Tan et al., 2015; Tocker et al., 2017; Bassett et al., 2018). Here, attractor-like coherence refers to relative phase relationships and coupling organization resembling an attractor network; it does not imply that the system already possesses a mature ring attractor in early development (Bjerknes et al., 2015; Bassett et al., 2018; Angelaki & Laurens, 2020). This raises a central question: how can the HD system develop internal organization before stable sensory anchoring is established, and how does this early organization later develop into the mature circuit for directional maintenance and velocity updating (Bassett et al., 2018; Tan et al., 2015; Taube, 2007; Angelaki & Laurens, 2020)?

It is important to note that direct evidence for early HD coherence comes mainly from downstream regions (Tan et al., 2015; Bjerknes et al., 2015; Bassett et al., 2018) rather than from direct recordings in the more proximal LMN–DTN loop. We therefore do not infer from the available data that LMN–DTN already expresses mature dynamics before eye opening. Rather, if downstream regions already show marked attractor-like internal coherence, some more central HD-generating module upstream should already possess the capacity to generate ordered internal dynamics. We focus on the lateral mammillary nucleus and dorsal tegmental nucleus (LMN–DTN) because anatomical studies (Mehlman et al., 2021), lesion (Blair et al., 1998; Bassett et al., 2007) and circuit analyses (Sharp et al., 2001a; Taube, 2007; Clark & Taube, 2012), and physiological studies (Sharp et al., 2001b; Bassett & Taube, 2001; Taube et al., 2025) consistently indicate that this loop is closely related to directional maintenance (Taube, 2007; Clark & Taube, 2012) and angular head velocity updating (Stackman & Taube, 1997; Sharp et al., 2001b; Bassett & Taube, 2001; Taube et al., 2025).

Previous models have addressed aspects of HD circuit formation (Stringer & Rolls, 2006; Hahnloser, 2003; Vafidis et al., 2022; Stentiford et al., 2022). Classical ring-attractor models account for single-bump maintenance (Zhang, 1996; Page et al., 2018), velocity-driven translation (Song & Wang, 2005; Page et al., 2018), and path integration in mature circuits (Zhang, 1996; Song & Wang, 2005; Stringer & Rolls, 2006), but usually begin from circuits whose core structure is already in place (Zhang, 1996; Song & Wang, 2005; Stringer & Rolls, 2006; Page et al., 2018). More recent studies have considered learning, self-organization, or sensory calibration (Hahnloser, 2003; Vafidis et al., 2022; Stentiford et al., 2022; Bicanski & Burgess, 2016). However, such models generally either focus on tuning mature circuits, or rely on pre-existing ring topology, visual teacher signals, or relatively abstract self-organizing rules (Hahnloser, 2003; Stringer & Rolls, 2006; Vafidis et al., 2022; Page et al., 2018; Bicanski & Burgess, 2016). To our knowledge, they do not directly address whether a more central LMN–DTN circuit could first self-organize an early internal structure through endogenous structured dynamics and local plasticity, and only later develop into the mature HD–AHV circuit.

In this context, we draw on a broader developmental principle: before mature externally driven responses are established, structured spontaneous activity often provides a statistical scaffold for later circuit assembly (Martini et al., 2021; Wong, 1999; Clause et al., 2014). Results from the visual (Wong, 1999; Ackman et al., 2012), auditory (Jones et al., 2007; Clause et al., 2014), and somatosensory systems (Khazipov et al., 2004; Dooley et al., 2020) together suggest that such activity is not merely noise, but may serve as a wiring template by providing repeatable temporal correlations and coactivation statistics for local plasticity rules (Ackman et al., 2012; Clause et al., 2014; Khazipov et al., 2004). Among the possible forms of early endogenous activity, we focus specifically on travelling-wave or synfire-chain-like propagation dynamics. Such dynamics can arise naturally in random recurrent networks under weak heterogeneity and refractory-like mechanisms (Meijer & Coombes, 2014), and therefore need not be imposed as an external template (Weistuch et al., 2021). Compared with synchronous bursts (Abeles, 1991; Herrmann et al., 1995; Diesmann et al., 1999) or unstructured fluctuations, they also provide a stable and directional temporal ordering of activity (Abeles, 2009; Haß et al., 2008; Morrison et al., 2008), making them especially suitable as a substrate for local plasticity to build phase-ordered structure and direction-selective update pathways (Butz et al., 2009; Zheng & Triesch, 2014; Bennett & Bair, 2015).

Based on this idea, we propose that the HD system may pass through a dynamics-first stage. A randomly connected recurrent LMN network first generates repeatable travelling waves (Meijer & Coombes, 2014; Weistuch et al., 2021), which we interpret as a synfire-chain-like regime of sequential propagation (Abeles, 1991, 2009; Herrmann et al., 1995; Diesmann et al., 1999) rather than a mature ring attractor. STDP-like and structural plasticity then write these temporal statistics into recurrent connectivity, producing a locally enhanced but imperfectly symmetric structure (Morrison et al., 2008; Butz et al., 2009; Zheng & Triesch, 2014; Bennett & Bair, 2015). This recurrent-structure learning is followed by direction-selective shift-feedback learning in the DTN*→ *LMN pathway. Once long-range inhibition is recruited later in development, the system transitions from propagating-wave dynamics to a stable single-bump attractor. When visual cues become available, they no longer construct the internal manifold, but instead act as a calibration signal that adjusts overall integration gain (Goodridge et al., 1998; Yoder et al., 2015) and improves path integration (Hahnloser, 2003; Bicanski & Burgess, 2016; Vafidis et al., 2022; Stentiford et al., 2022).

## Methods

### Model overview and developmental stages

We constructed a developmental rate-based model of the LMN–DTN head direction circuit. The model contains a recurrent excitatory LMN population ($N_L=400$), which carries the population representation of head direction, and two DTN conjunctive populations corresponding to clockwise (CW) and counterclockwise (CCW) velocity channels (each with $N_D=400$), which receive angular velocity signals and drive bump translation (Clark & Taube, 2012).

Plastic connections are the recurrent LMN matrix $\vec {W}$ and the DTN*→ *LMN feedback matrices $\vec {W}_{DL}^{\textrm{cw}}$ and $\vec {W}_{DL}^{\textrm{ccw}}$. Fixed components are the local LMN*→ *DTN readout kernels $\vec {W}_{LD}^{\textrm{cw}}$ and $\vec {W}_{LD}^{\textrm{ccw}}$, and a long-range inhibition kernel $\vec {K}_{\textrm{inh}}$ that depends only on distance along the ring.

The model develops through four stages. In the spontaneous-dynamics stage, the random LMN network evolves without learning, long-range inhibition, or external input, and generates travelling waves through relative refractory dynamics (Meijer & Coombes, 2014). In the structure-formation stage, STDP-like and structural plasticity first consolidate the temporal statistics of these waves into locally enhanced recurrent connectivity, followed by direction-selective shift-feedback learning in the DTN*→ *LMN pathway (Bennett & Bair, 2015). In the attractor-formation stage, long-range inhibition is recruited and converts travelling-wave dynamics into a stable single-bump attractor (Amari, 1977). In the sensory-calibration stage, corresponding to the period after eye opening, visual input calibrates the global path-integration gain $g_{\textrm{DTN}}$ (Yoder et al., 2015).

**Fig. 1:**
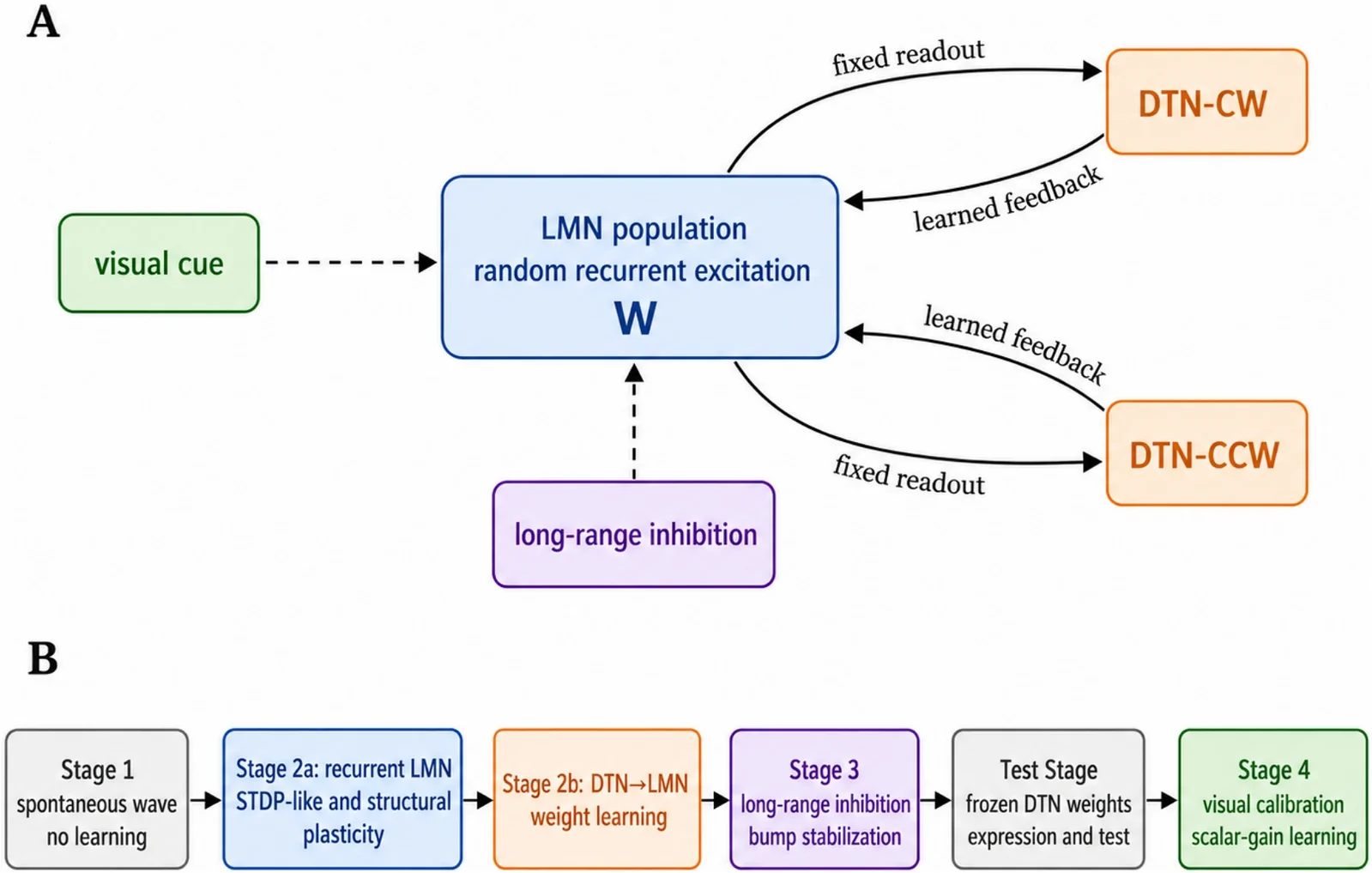
**Circuit architecture and staged protocol** **A**, The recurrent LMN population receives long-range inhibition and visual input and is coupled to clockwise (CW) and counterclockwise (CCW) DTN channels through fixed LMN*→ *DTN readouts and learned DTN*→ *LMN feedback. **B**, The developmental sequence separates learning from subsequent expression. Stage 2 comprises two consecutive substages: recurrent LMN plasticity occurs first in Stage 2a, followed immediately by DTN*→ *LMN weight learning in Stage 2b while feedback expression remains disabled. Long-range inhibition is recruited in Stage 3, after which the frozen feedback matrices are first expressed and tested during the separate Test Stage. The Test Stage is an evaluation phase rather than an additional developmental stage. During Stage 4, all synaptic matrices remain fixed and only the scalar DTN expression gain is calibrated. Relative refractoriness and early homogeneous global inhibition are intrinsic components of LMN activity and are not shown in the schematic

These four stages are proposed not as four directly established biological epochs of LMN–DTN development, but as a developmental–computational framework constrained by both experimental observations and model-internal functional dependence. Developmental data from downstream regions such as ADN and PoS/PrSd show that internal HD coherence emerges before stable sensory anchoring, whereas absolute directional tuning remains unstable before eye opening (Tan et al., 2015; Bjerknes et al., 2015; Bassett et al., 2018; Tocker et al., 2017). Because these observations come from downstream structures rather than directly from LMN–DTN, we do not take them to imply that the LMN–DTN loop already exhibits mature attractor dynamics at this stage. Instead, they motivate the inference that some upstream HD-generating substrate must already support ordered internal dynamics. We focus on LMN–DTN because convergent anatomical, lesion, circuit, and physiological evidence identifies this loop as a plausible substrate for directional maintenance and angular-velocity updating (Sharp et al., 2001b; Taube et al., 2025). Within the model, the ordering of the stages is further constrained by computation: repeatable endogenous dynamics must precede plastic consolidation (Martini et al., 2021), partially organized recurrent structure must precede long-range stabilization into a bump attractor (Amari, 1977; Page et al., 2018), and a usable integrator must precede late sensory calibration (Yoder et al., 2015). The four stages therefore represent four computationally distinct but developmentally ordered regimes, rather than four directly observed biological periods of LMN–DTN maturation (Fig. 1).

Accordingly, the Results are organized around the corresponding questions of preconditions for wave emergence, structural consequences of plasticity, convergence to attractor dynamics, functional consequences for path integration, and quantitative evaluation of the calibrated circuit.

### LMN population dynamics

LMN activity $\vec {x}_t\in \mathbb {R}_{\ge 0}^{N_L}$ is updated by

1 $$\begin{aligned} \vec {x}_t=(1-\lambda )\vec {x}_{t-1}+\lambda \,\sigma (\vec {h}_t), \end{aligned}$$

where $\lambda \in (0,1]$ is the leak coefficient and

2 $$\begin{aligned} \sigma (\vec {h}_t)=\frac{c}{1+\exp \left\{ -\beta (\vec {h}_t-\theta )\right\} } \end{aligned}$$

is a bounded sigmoid with slope *β *, threshold *θ *, and saturation constant *c*.

The total LMN input is

3 $$\begin{aligned} \vec {h}_t= \vec {W}\vec {x}_{t-1} -G_{\textrm{inh}}\vec {K}_{\textrm{inh}}\vec {x}_{t-1} +\vec {I}^{\textrm{DTN}}_t +\boldsymbol{\xi }^{\textrm{noise}}_t +\vec {I}^{\textrm{inh}}_t +\vec {I}^{\textrm{VIS}}_t +\vec {I}^{\textrm{refr}}_t. \end{aligned}$$

Here $\vec {W}$ is a sparse random excitatory matrix with connection probability *p=0.3* and no imposed structure; $G_{\textrm{inh}}=0$ before the attractor-formation stage; $\vec {I}^{\textrm{DTN}}_t$ is DTN feedback; $\boldsymbol{\xi }^{\textrm{noise}}_t$ is Gaussian noise; $\vec {I}^{\textrm{inh}}_t$ denotes a global inhibitory input, with the same inhibitory contribution applied to every neuron; and $\vec {I}^{\textrm{VIS}}_t$ is visual input used only during calibration. $\vec {I}^{\textrm{refr}}_t$ is a relative refractory penalty term that transiently suppresses recently active neurons and is the key mechanism supporting wave generation.

### DTN pathway and velocity-gated feedback

DTN populations receive fixed local readout from LMN. For analysis and visualization we define phase-ordered LMN activity $\vec {x}^{\textrm{ord}}_t$, noting that this ordering is not used by the network itself. DTN readout is given by

4 $$\begin{aligned} \vec {y}^{\textrm{cw}}_t={{\,\textrm{ReLU}\,}}\left( \vec {W}^{\textrm{cw}}_{LD}\vec {x}^{\textrm{ord}}_t\right) ,\qquad \vec {y}^{\textrm{ccw}}_t={{\,\textrm{ReLU}\,}}\left( \vec {W}^{\textrm{ccw}}_{LD}\vec {x}^{\textrm{ord}}_t\right) , \end{aligned}$$

where $\vec {W}_{LD}^{\mathrm {cw/ccw}}$ are fixed narrow Gaussian kernels corresponding to local HD tuning in DTN (Clark & Taube, 2012).

Angular velocity input $\omega _t$ gates the two channels as

5 $$\begin{aligned} \vec {y}^{\textrm{cw,drive}}_t=\vec {y}^{\textrm{cw}}_t\cdot \max (\omega _t,0),\qquad \vec {y}^{\textrm{ccw,drive}}_t=\vec {y}^{\textrm{ccw}}_t\cdot \max (-\omega _t,0), \end{aligned}$$

and DTN*→ *LMN feedback is

6 $$\begin{aligned} \vec {I}^{\textrm{DTN}}_t= g_{\textrm{DTN}} \left( \vec {W}^{\textrm{cw}}_{DL}\vec {y}^{\textrm{cw,drive}}_t+ \vec {W}^{\textrm{ccw}}_{DL}\vec {y}^{\textrm{ccw,drive}}_t \right) . \end{aligned}$$

$g_{\textrm{DTN}}$ is a global gain parameter adjusted during late calibration.

### Plasticity rules

#### LMN recurrent connectivity

Recurrent LMN connectivity is learned by combining STDP-like and structural plasticity. To capture temporal correlations in a rate-based model, we define presynaptic and postsynaptic eligibility traces

7 $$\begin{aligned} \vec {e}_{\textrm{pre}}(t) = \left( 1 - \frac{1}{\tau _{\textrm{pre}}}\right) \vec {e}_{\textrm{pre}}(t-1) + \frac{1}{\tau _{\textrm{pre}}}\vec {x}(t-1), \end{aligned}$$

8 $$\begin{aligned} \vec {e}_{\textrm{post}}(t) = \left( 1 - \frac{1}{\tau _{\textrm{post}}}\right) \vec {e}_{\textrm{post}}(t-1) + \frac{1}{\tau _{\textrm{post}}}\vec {x}(t), \end{aligned}$$

where $\tau _{\textrm{pre}}$ and $\tau _{\textrm{post}}$ set the temporal window of wave-related correlations. The STDP-like update is

9 $$\begin{aligned} \Delta \vec {W}_{\textrm{stdp}}(t) = \eta \left[ \vec {x}(t)\vec {e}_{\textrm{pre}}(t)^\top - A_- \vec {e}_{\textrm{post}}(t)\vec {x}(t-1)^\top \right] \odot \textbf{M}(t), \end{aligned}$$

followed by

10 $$\begin{aligned} \vec {W}(t) \leftarrow \vec {W}(t-1) + \Delta \vec {W}_{\textrm{stdp}}(t), \end{aligned}$$

where *η * is the learning rate, $A_-$ scales depression, $\textbf{M}(t)$ is the structural mask, and *⊙ * denotes the Hadamard (element-wise) product, such that plasticity updates are restricted to entries permitted by the structural mask. After each update, weights are constrained to remain non-negative, upper bounded, and row-normalized to a fixed target sum. Recurrent learning is turned off during the final part of the structure-formation stage.

In biological systems, synaptic reorganization involves not only changes in synaptic strength but also structural plasticity. Accordingly, we incorporate an additional structural plasticity mechanism based on pairwise coactivation (Butz et al., 2009). At regular intervals during the structure-formation stage, we update an exponential moving average of pairwise coactivation as

11 $$\begin{aligned} \textbf{C}_{\textrm{ema}}(t) = (1 - \alpha )\textbf{C}_{\textrm{ema}}(t-1) + \alpha \left[ \vec {x}(t)\vec {x}(t)^\top - \textrm{diag}\left( \vec {x}(t)\vec {x}(t)^\top \right) \right] , \end{aligned}$$

where $\vec {x}(t)\in \mathbb {R}^{N_L}$ is the LMN activity vector at time *t*, $\textbf{C}_{\textrm{ema}}(t)\in \mathbb {R}^{N_L\times N_L}$ is the EMA coactivation matrix, *α * is the update rate, and the diagonal is removed to exclude self-connections. Structural plasticity consists of three operations. First, all weights may undergo optional global decay:

12 $$\begin{aligned} \vec {W}(t) \leftarrow (1 - \lambda _{\textrm{decay}})\vec {W}(t), \end{aligned}$$

where $\vec {W}(t)\in \mathbb {R}^{N_L\times N_L}$ is the LMN recurrent weight matrix and $\lambda _{\textrm{decay}}$ is the decay factor. Second, existing connections are pruned when either weight or correlation falls below threshold:

13 $$\begin{aligned} \text {if}\ W_{ij}(t)< w_{\textrm{min}} \ \text {or}\ C_{\textrm{ema},ij}(t) < c_{\textrm{min}}, \quad \Rightarrow \quad M_{ij}(t) \leftarrow 0,\ W_{ij}(t) \leftarrow 0, \end{aligned}$$

where $W_{ij}(t)$ is the weight from presynaptic neuron *j* to postsynaptic neuron *i*, $M_{ij}(t)\in \{0,1\}$ is the corresponding structural mask entry, $w_{\textrm{min}}$ is the pruning threshold for weight, and $c_{\textrm{min}}$ is the pruning threshold for coactivation. Third, for each postsynaptic neuron *i*, up to $k_{\textrm{add}}$ previously absent presynaptic connections with the highest coactivation scores are added. Let $\mathcal {S}_i(t)$ denote the corresponding set of selected presynaptic indices:

14a $$\begin{aligned} \mathcal {S}_i(t) \subseteq \left\{ j \mid M_{ij}(t)=0\right\} , \qquad \left| \mathcal {S}_i(t)\right| \le k_{\textrm{add}}. \end{aligned}$$

For each $j\in \mathcal {S}_i(t)$, the new connection is added according to

14b $$\begin{aligned} W_{ij}(t) \leftarrow W_{ij}(t) + w_{\textrm{new}} \cdot g(C_{\textrm{ema},ij}(t)) \cdot n(i,j), \qquad M_{ij}(t)\leftarrow 1, j\in \mathcal {S}_i(t). \end{aligned}$$

Here, $k_{\textrm{add}}$ is the maximum number of new presynaptic partners added per postsynaptic neuron at one structural update, $w_{\textrm{new}}$ sets the initial weight scale of newly added connections, *g(· )* normalizes the correlation score, and *n*(*i*, *j*) is an activity-derived phase-neighbor factor.

The phase-neighbor factor is calculated from functional phases inferred from spontaneous travelling-wave activity. Before structural learning, activity from a stable travelling-wave interval is projected into a low-dimensional population space, and the phase of the resulting closed population trajectory is estimated. Each LMN unit is then assigned a functional phase $\phi _i\in (-\pi ,\pi ]$ according to the relative phase of its activity along this trajectory. The resulting circular ordering is therefore inferred from the emergent travelling-wave dynamics rather than assigned from neuronal indices or a preconfigured anatomical arrangement, and it is held fixed during subsequent structural learning. We define

14c $$\begin{aligned} \begin{gathered} d_{\textrm{circ}}(\phi _i,\phi _j) = \left| {\text {wrap}}(\phi _i-\phi _j) \right| ,\\ n(i,j) = \exp \left[ -\frac{ d_{\textrm{circ}}(\phi _i,\phi _j)^2 }{ 2\sigma _n^2 } \right] , \end{gathered} \end{aligned}$$

where ${\text {wrap}}(\cdot )$ maps an angular difference to $(-\pi ,\pi ]$, $d_{\textrm{circ}}(\phi _i,\phi _j)\in [0,\pi ]$ is the shortest circular phase distance, and $\sigma _n$ controls the width of the phase-neighbor growth bias. Thus, *n*(*i*, *j*) is a Gaussian kernel of shortest circular phase distance that preferentially promotes growth between units with nearby activity-derived phases. After each structural update, the same amplitude and row-normalization constraints are applied.

#### DTN*→ *LMN feedback

DTN*→ *LMN feedback is learned by a direction-gated outer-product rule. Let $\vec {x}_t^{\textrm{ord}}$ be the phase-ordered LMN activity and $\vec {y}^{\textrm{cw}}_{t-1}, \vec {y}^{\textrm{ccw}}_{t-1}$ the DTN activities at the previous step. For the CW channel,

15 $$\begin{aligned} \Delta \vec {W}_{DL}^{\textrm{cw}}(t) = \eta _{DL} |\hat{\omega }(t-1)| \left( \vec {x}_t^{\textrm{ord}} \left[ \vec {y}^{\textrm{cw}}_{t-1}\right] ^\top - A_{DL}^- \vec {x}_{t-2}^{\textrm{ord}} \left[ \vec {y}^{\textrm{cw}}_{t-1}\right] ^\top \right) \odot \textbf{M}^{DL}, \end{aligned}$$

and the CCW channel is updated analogously,

16 $$\begin{aligned} \Delta \vec {W}_{DL}^{\textrm{ccw}}(t) = \eta _{DL} |\hat{\omega }(t-1)| \left( \vec {x}_t^{\textrm{ord}} \left[ \vec {y}^{\textrm{ccw}}_{t-1}\right] ^\top - A_{DL}^- \vec {x}_{t-2}^{\textrm{ord}} \left[ \vec {y}^{\textrm{ccw}}_{t-1}\right] ^\top \right) \odot \textbf{M}^{DL}. \end{aligned}$$

Here $\eta _{DL}$ is the learning rate, $|\hat{\omega }(t-1)|$ scales learning by propagation speed, $A_{DL}^-$ is the subtractive coefficient, and $\textbf{M}^{DL}$ is the fixed DTN*→ *LMN mask. In the actual simulation, learning uses the original neuron indices; phase ordering is used only to describe the learned push–pull structure. Weights are updated as

17 $$\begin{aligned} \vec {W}_{DL}(t) \leftarrow \vec {W}_{DL}(t-1) + \Delta \vec {W}_{DL}(t), \end{aligned}$$

and then clipped to a symmetric bounded range. Because spontaneous travelling waves are typically unidirectional, we optionally mirror the learning signal across channels so that both CW and CCW connections can be trained from the same wave statistics (Song & Wang, 2005).

### Phase ordering and direction decoding

Because neuron indices have no geometric meaning at initialization, we estimate a fixed phase-ordering permutation *π * from a time window containing stable travelling waves and define

18 $$\begin{aligned} \vec {x}^{\textrm{ord}}_t=\vec {x}_t[\pi ]. \end{aligned}$$

This activity-derived ordering is used to define the phase-neighbour bias during structural learning and is also used for analysis, visualization, and decoding. It is inferred from spontaneous travelling-wave activity rather than assigned at initialization, and therefore does not impose a preconfigured anatomical topology. Internal head direction is decoded by a population vector:

19 $$\begin{aligned} \hat{\theta }_t= \arg \left( \sum _{i=0}^{N_L-1} x^{\textrm{ord}}_{t,i} e^{\textrm{j}\theta _i} \right) , \qquad \theta _i=\frac{2\pi i}{N_L}, \end{aligned}$$

and directional error is

20 $$\begin{aligned} e_t^\theta = {{\,\textrm{wrap}\,}}\left( \hat{\theta }_t-\theta _t^{\textrm{true}}\right) , \end{aligned}$$

where ${{\,\textrm{wrap}\,}}(\cdot )$ is the circular wrapping operation defined above. Directional errors are converted from radians to degrees for reporting and visualization.

### Visual calibration and quantitative measures

The visual calibration stage has two substages. In substage 1, visual teacher input and vestibular drive are both present, and the global gain $g_{\textrm{DTN}}$ is adjusted according to the mismatch between internally estimated and externally referenced angular velocity. In substage 2, visual input is removed, all learning is frozen, and path integration is tested under purely vestibular drive.

The visual input to LMN cell *i* was implemented as a peak-normalized Gaussian kernel of the shortest circular distance,

21 $$\begin{aligned} I^{\textrm{VIS}}_i(t) = A_{\textrm{VIS}}\,s(t) \frac{ \exp \!\left[ -d_{\textrm{circ}}(\phi _i,\theta _{\textrm{VIS}}(t))^2/ (2\sigma _{\textrm{VIS}}^2) \right] }{ \max _j \exp \!\left[ -d_{\textrm{circ}}(\phi _j,\theta _{\textrm{VIS}}(t))^2/ (2\sigma _{\textrm{VIS}}^2) \right] }, \end{aligned}$$

where $A_{\textrm{VIS}}$ is the peak input amplitude, $\sigma _{\textrm{VIS}}$ is the angular width, $\phi _i$ is the activity-derived functional phase of unit *i*, and $\theta _{\textrm{VIS}}(t)$ is the visual-cue centre. For angular variables *a* and *b*, the shortest circular distance is defined as

22 $$\begin{aligned} d_{\textrm{circ}}(a,b) = \left| {\text {wrap}}(a-b) \right| \in [0,\pi ], \end{aligned}$$

where ${\text {wrap}}(\cdot )$ is the circular wrapping operation defined above. The temporal envelope *s*(*t*) was sustained, exponentially decaying, or intermittently switched on and off. The effects of input amplitude, width, temporal profile, and presentation duration were examined separately.

To quantify development and final function, we used five classes of measures: wave-quality measures (trajectory closure, $R^2$, $\textrm{CV}_r$, and $\textrm{CV}_\omega$; stable waves were defined by $R^{2}\ge 0.9$, $\textrm{CV}_{\omega }\le 0.2$, and $\textrm{CV}_{r}\le 0.30$), connectivity-structure measures, attractor-stability measures, feedback-connection measures, and path-integration measures.

Here, $\textrm{CV}_{\omega }$ is the coefficient of variation of instantaneous phase velocity, with smaller values indicating more uniform wave propagation, whereas $\textrm{CV}_{r}$ is the coefficient of variation of the PC1–PC2 trajectory radius, with smaller values indicating greater radial stability. The post-learning local_ratio is the ratio of mean near-neighbour recurrent weight to mean distant recurrent weight, so larger values indicate stronger local connectivity. The symmetry_corr is the correlation between reciprocal weights $W_{ij}$ and $W_{ji}$, with values closer to one indicating greater bidirectional symmetry. Finally, dist_slope is the fitted slope of mean recurrent weight against circular phase distance; more negative values indicate stronger distance-dependent decay and a more pronounced local connectivity band.

Unless otherwise stated, probabilities, across-run means, and variability estimates were computed from *n=10* independently initialized simulations. Panels showing representative trajectories, activity profiles, or weight matrices depict single simulations for visualization and are not used to estimate across-run uncertainty. Repeated onset-state or replay analyses within a fixed learned network are reported separately as within-network replays rather than as independent network realizations. Likewise, late visual-calibration analyses that vary the sensory signal within an already learned and frozen network use the stated numbers of calibration episodes and cue-off noise realizations, because these analyses quantify within-network calibration robustness rather than variability across independently developed networks.

Unless otherwise stated, simulations used the reference parameter values summarized in Table 1. These values correspond to the baseline conditions indicated by dotted reference lines, circled “B” markers, or stars in the main-text and Appendix parameter-sensitivity figures; parameters varied in a given analysis are listed in the corresponding figure caption.

**Table 1:** Main reference parameter values used in the simulations and parameter-sensitivity analyses

Component	Parameter	Reference value	Definition or use
Network size	$N_L$	400	Number of LMN units.
Network size	$N_D$	400 per channel	Number of units in each CW and CCW DTN population.
Initial connectivity	*p*	0.30	Initial LMN recurrent connection probability.
Stage 1 dynamics	g_inh	0.8	Strength of the early homogeneous global-inhibitory input (code-style label in Fig. 4).
Stage 1 dynamics	ref_steps	40 steps	Relative-refractory duration.
Stage 1 dynamics	ref_soft_amp	2.0	Relative-refractory strength.
Noise	$\mu _{\textrm{noise}}$	0	Mean of the Gaussian noise distribution.
Noise	$\sigma _{\textrm{noise}}$	0.01	Standard deviation of the Gaussian noise distribution.
Recurrent plasticity	*η *	$3\times 10^{-4}$	STDP-like learning rate.
Recurrent plasticity	$A_-$	0.6	Depression scaling factor.
Recurrent plasticity	$\tau _{\textrm{pre}}=\tau _{\textrm{post}}$	20 steps	Eligibility-trace time constants.
Structural growth	$k_{\textrm{add}}$	150	Maximum number of new presynaptic partners added per postsynaptic unit at each structural update.
Coactivation estimate	*α *	$3\times 10^{-3}$	EMA update rate for pairwise coactivation.
New-edge strength	$w_{\textrm{new}}$	0.30	Multiplicative scale factor determining the initial weight of newly added connections.
Structural decay	$\lambda _{\textrm{decay}}$	0.01	Global recurrent-weight decay factor applied at a structural update.
Pruning	$w_{\textrm{min}}$	0.10	Weight-pruning threshold.
Pruning	$c_{\textrm{min}}$	0.005	Coactivation-pruning threshold.
Visual calibration	$A_{\textrm{VIS}}$	1.0 selected	Peak visual-input amplitude. The selected value is the filled-star condition used for the temporal-profile analyses; the original default is the outlined-star condition in Appendix Fig. 14.
		2.0 original default	
Visual calibration	$\sigma _{\textrm{VIS}}$	$12.6^{\circ }$ selected	Circular-Gaussian visual-input width, using the same filled-star and outlined-star conventions as above.
		$50.4^{\circ }$ original default	

## Results

### Spontaneous travelling waves emerge in random LMN networks

**Fig. 2:**
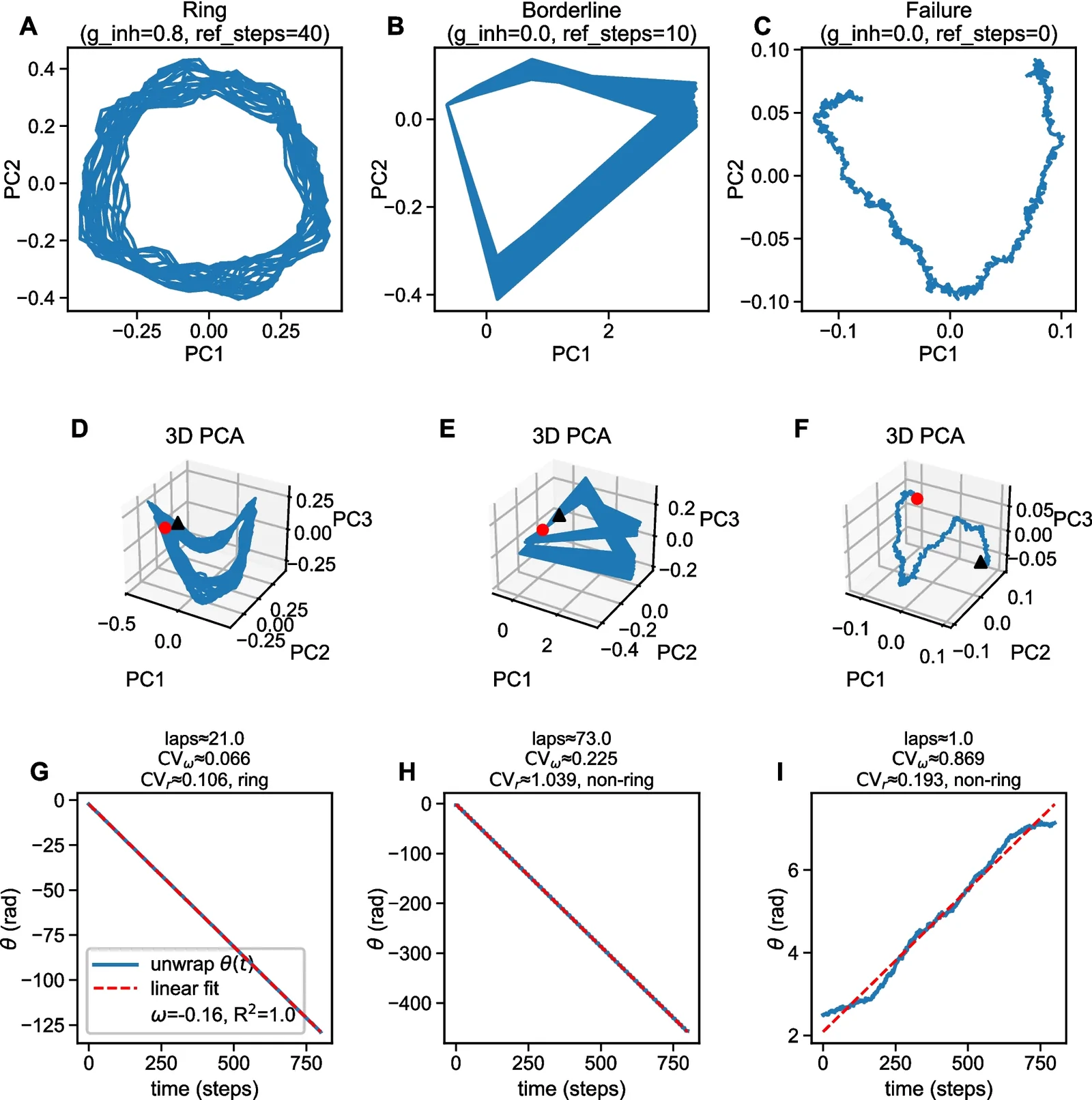
Representative spontaneous LMN dynamics before learning **A–C**, PC1–PC2 trajectories for the stable Ring, Borderline, and Failure regimes, which differ in global-inhibition strength and relative-refractory duration as indicated above each panel. **D–F**, corresponding three-dimensional PCA trajectories; red circles and black triangles mark the beginning and end of the analysed interval, respectively. **G–I**, unwrapped population phase *θ (t)* over the same interval; red dashed lines show linear fits to phase progression. $n_{\textrm{laps}}$ is the number of completed propagation cycles, $\textrm{CV}_{\omega }$ is the coefficient of variation of instantaneous phase velocity, and $\textrm{CV}_{r}$ is the coefficient of variation of the PC1–PC2 radius. Each column shows one representative simulation analysed over the late pre-learning interval

We first asked whether a randomly connected LMN network, in the absence of preconfigured structure, learning, or external input, could spontaneously generate stable travelling waves. As shown in Fig. 2, the pre-learning network exhibited three typical dynamical regimes. In the stable-wave regime, the PC1/PC2 trajectory formed a smooth closed loop, the unwrapped phase advanced approximately linearly, and propagation continued for multiple cycles. In the borderline regime, periodicity remained but the trajectory was distorted and both propagation speed and activity amplitude fluctuated more strongly. In the failure regime, the trajectory no longer formed a loop and phase progression lost regularity.

**Fig. 3:**
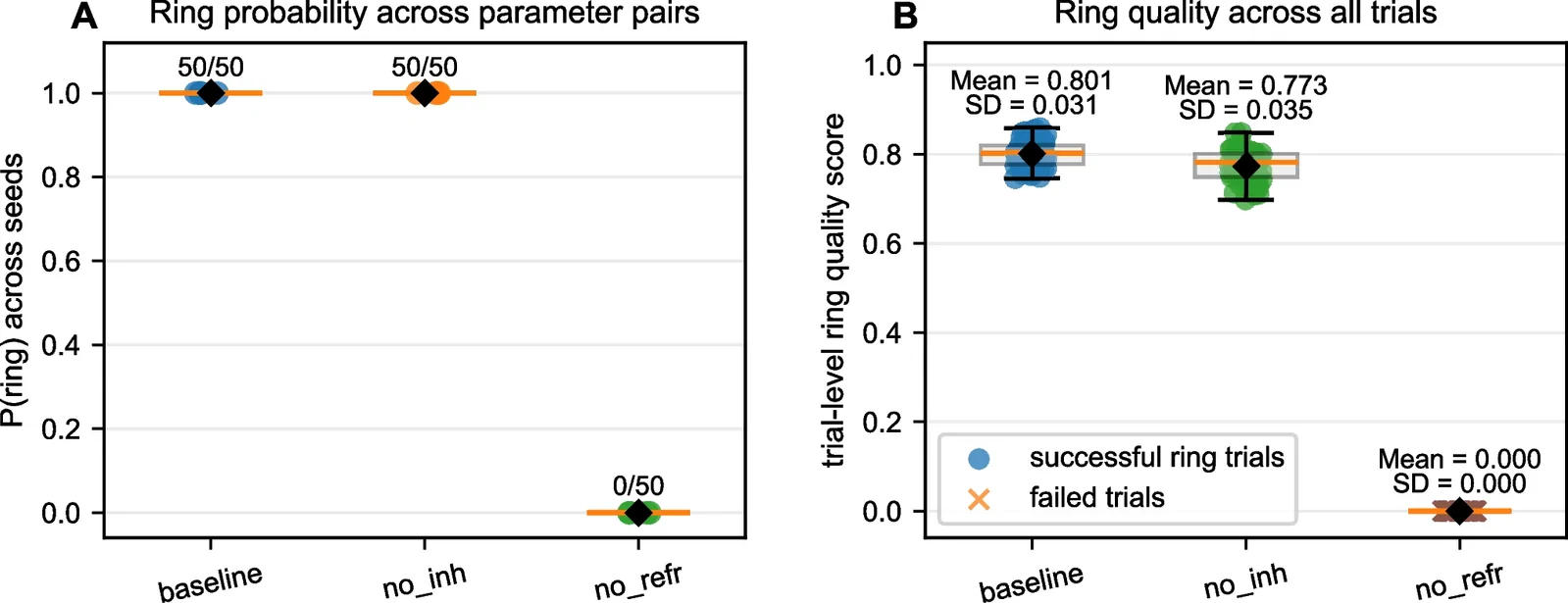
Relative refractoriness is required for spontaneous travelling-wave generation Three conditions were tested across five stable ring-supporting parameter combinations, with *n=10* independently initialized simulation runs for each parameter combination. Thus, each condition comprised 50 independent runs in total. **A**, $P(\textit{ring})$, defined separately for each parameter combination as the fraction of the 10 runs classified as stable rings. Jittered points represent the five parameter combinations, boxes show the median and interquartile range across those combinations, black diamonds indicate the mean across parameter combinations, and numbers above each group give successful/total trials across all 50 runs for that condition. **B**, trial-level ring-quality scores for all 50 runs per condition; circles denote successful rings, whereas crosses denote failed trials, which were assigned a score of zero. The baseline retained both mechanisms, no inhibition removed global inhibition, and no refractoriness removed relative refractoriness. A run was classified as a stable ring when $R^{2}\ge 0.9$, $\textrm{CV}_{\omega }\le 0.2$, $\textrm{CV}_{r}\le 0.30$, and at least two complete propagation cycles were present

**Fig. 4:**
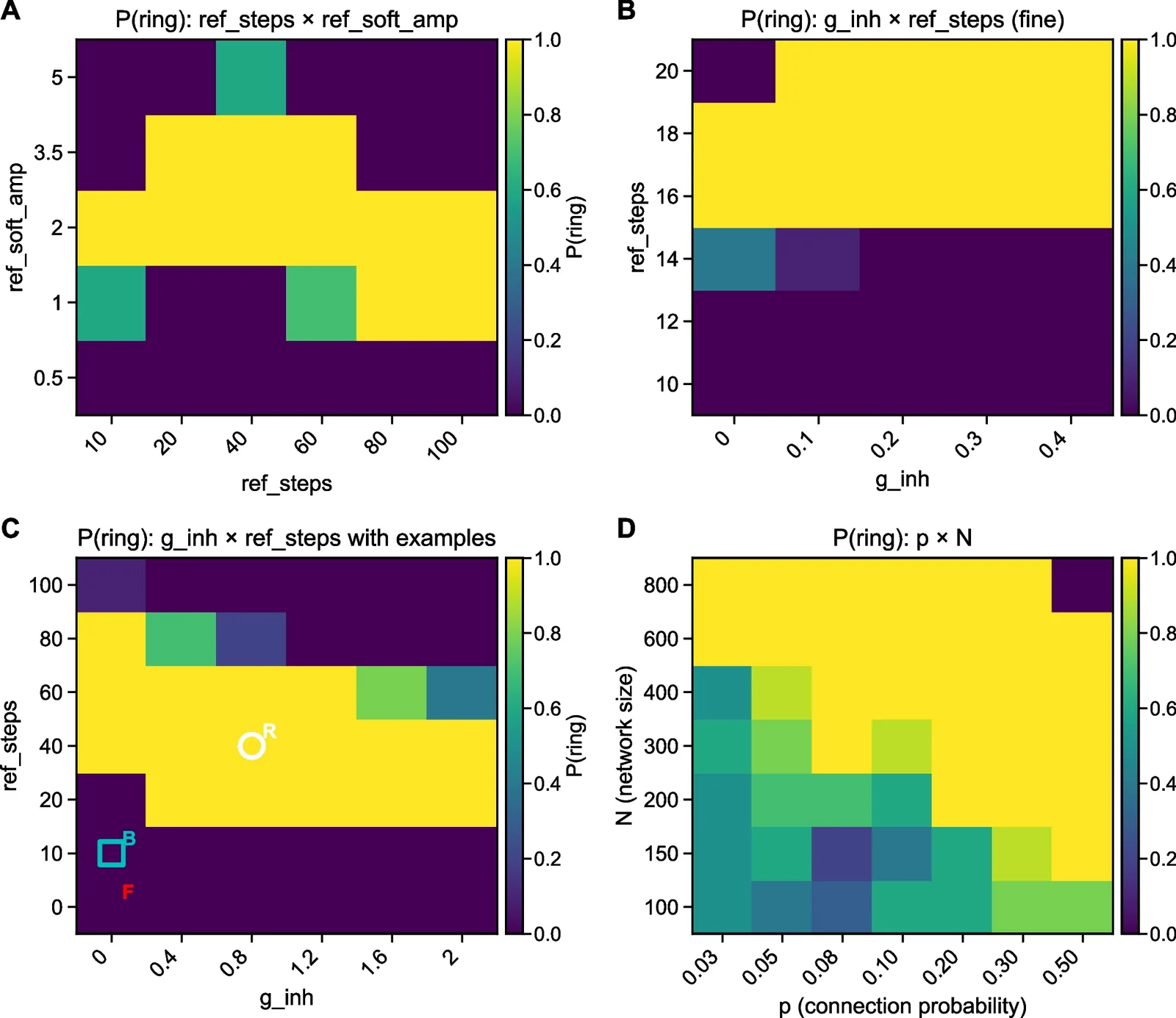
Travelling-wave generation is robust across broad parameter regions but depends jointly on relative refractoriness, global inhibition, connection probability, and network size Colour denotes $P(\textit{ring})$, defined at each parameter combination as the fraction of independent runs classified as stable rings. In the figure, the code-style labels ref_steps, ref_soft_amp, and g_inh denote relative-refractory duration, relative-refractory strength, and global-inhibition strength, respectively. **A**, scan of relative-refractory duration and strength, based on *n=10* independent runs per parameter combination. **B**, fine scan of global-inhibition strength and relative-refractory duration, based on *n=10* independent runs per parameter combination. **C**, broader scan of the same two parameters, based on *n=10* independent runs per parameter combination; R, B, and F mark the representative Ring, Borderline, and Failure regimes shown in Fig. 2. **D**, joint scan of initial random connection probability *p* and LMN network size $N_L$ (labelled *N* in the figure), based on *n=10* independent runs per parameter combination, with global-inhibition strength and relative-refractory duration fixed at their baseline values. Stable-wave generation was robust across a broad intermediate range of *p*, while smaller networks were less reliable and excessively dense connectivity reduced success at the largest network sizes. Panel D was restricted to the pre-learning stage because subsequent structural plasticity adds, prunes, and reweights connections, so the initial value of *p* is no longer a complete descriptor of the learned connectivity. A run was classified as a stable ring when $R^{2}\ge 0.9$, $\textrm{CV}_{\omega }\le 0.2$, $\textrm{CV}_{r}\le 0.30$, and at least two complete propagation cycles were present

Figure 3 shows that removing global inhibition did not abolish the wave, whereas removing relative refractoriness eliminated stable travelling waves. Wave generation therefore does not depend on mature inhibitory circuitry, but on the transient suppression of recently active neurons. Two-dimensional parameter scans further support this interpretation (Fig. 4): Stable travelling waves persisted across broad regions of relative-refractory duration and strength and global-inhibition strength. The additional scan of connection probability and network size showed that the reference value *p=0.30* was not a finely tuned requirement. Stable waves were obtained across a broad intermediate range of connection probabilities, particularly in larger networks, whereas smaller networks were less reliable and the densest tested connectivity reduced success in the largest networks. This scan was restricted to the pre-learning Stage 1 because, after wave formation, the subsequent plasticity rule adds, removes, and reweights synapses, such that the initial random connection probability no longer uniquely characterizes the learned network. Thus, stable travelling waves are an endogenous ordered dynamical state that can arise spontaneously in a random excitatory network and provide the temporal scaffold for later self-organization.

### Travelling waves scaffold recurrent self-organization

**Fig. 5:**
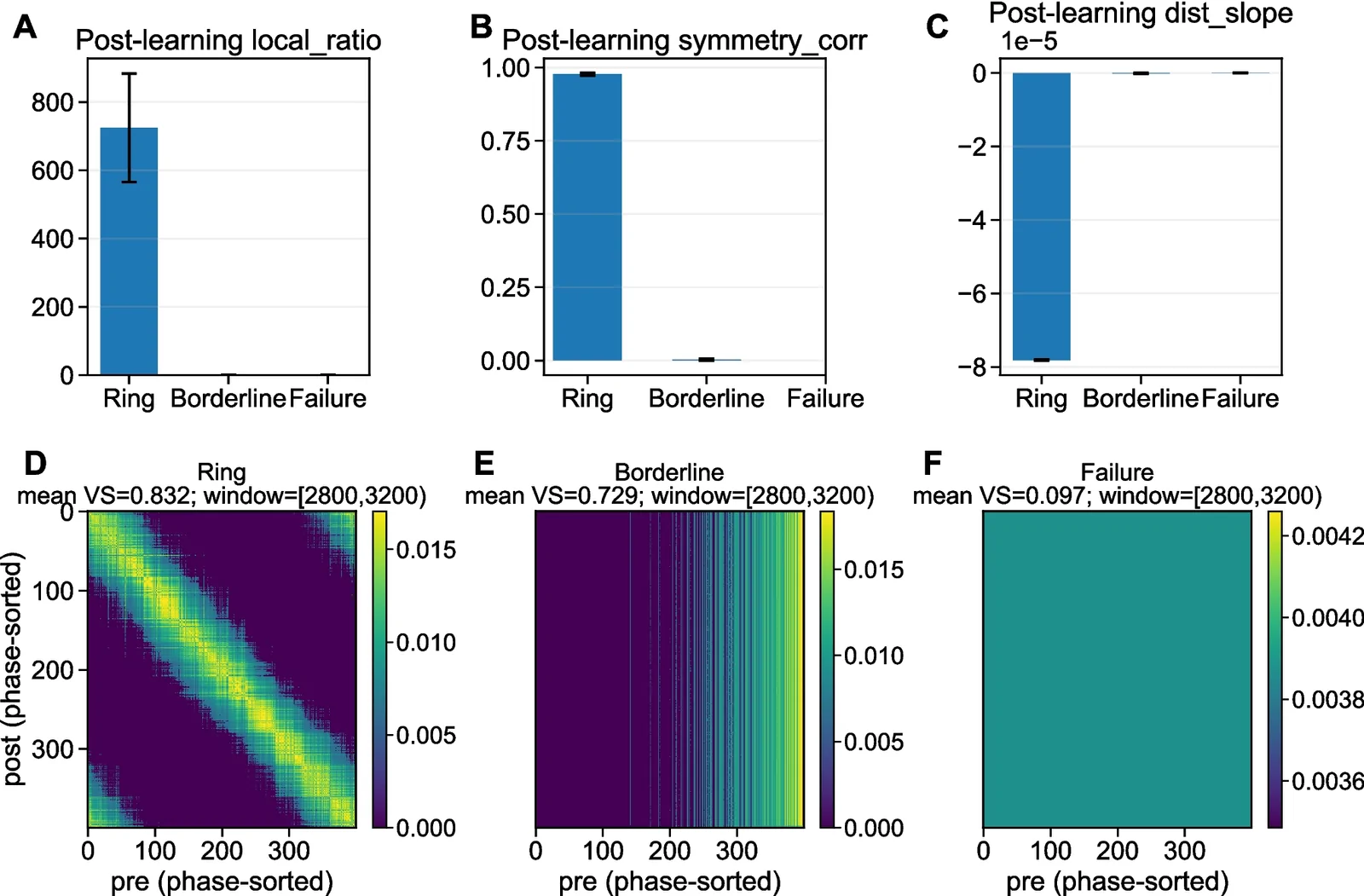
Post-learning recurrent structure depends on the quality of the pre-learning travelling wave **A–C**, post-learning locality, bidirectional symmetry, and distance-dependent weight slope in the Ring, Borderline, and Failure regimes. Bars show the mean across *n=10* independently initialized simulation runs, and error bars indicate mean ± 1 SD. Locality is the ratio of mean near-neighbour to distant recurrent weight; symmetry is the correlation between reciprocal connections; and the distance slope quantifies the change in mean recurrent weight with circular phase distance. **D–F**, representative recurrent matrices from the corresponding regimes, reordered by neuronal preferred phase; colour denotes recurrent weight. Mean VS denotes vector strength, averaged across LMN units, and quantifies the strength of phase tuning; larger values indicate more consistent activity around a preferred travelling-wave phase. All three regimes used the same plasticity rule and learning parameters, while their inhibition and refractory settings differed. Only the Ring regime developed a continuous local band with high symmetry and pronounced distance-dependent decay

We next asked whether these travelling waves could be written into recurrent connectivity by local plasticity. Figure 5 shows that post-learning structure depended strongly on pre-learning wave quality. Only in the stable-wave regime did the reordered recurrent matrix develop a clear local band along the main diagonal, together with strong nearest-neighbor enhancement, pronounced distance-dependent decay, and high bidirectional consistency. The borderline regime produced only coarse striping or one-sided bias, whereas the failure regime remained largely unstructured. Local plasticity therefore extracted consistent statistics only when the network first possessed a stable and repeatable propagation order.

In Fig. 5A, the elevated post-learning local_ratio in the Ring regime indicates that mean recurrent weights between units with nearby functional phases exceeded those between more distant units, consistent with preferential local excitation. In Fig. 5B, the high symmetry_corr indicates that strong connections were typically accompanied by strong reciprocal connections, consistent with a largely bidirectional local organization. In Fig. 5C, the negative dist_slope indicates that recurrent weight decreased with increasing circular phase distance, providing a direct signature of distance-dependent local excitation.

**Fig. 6:**
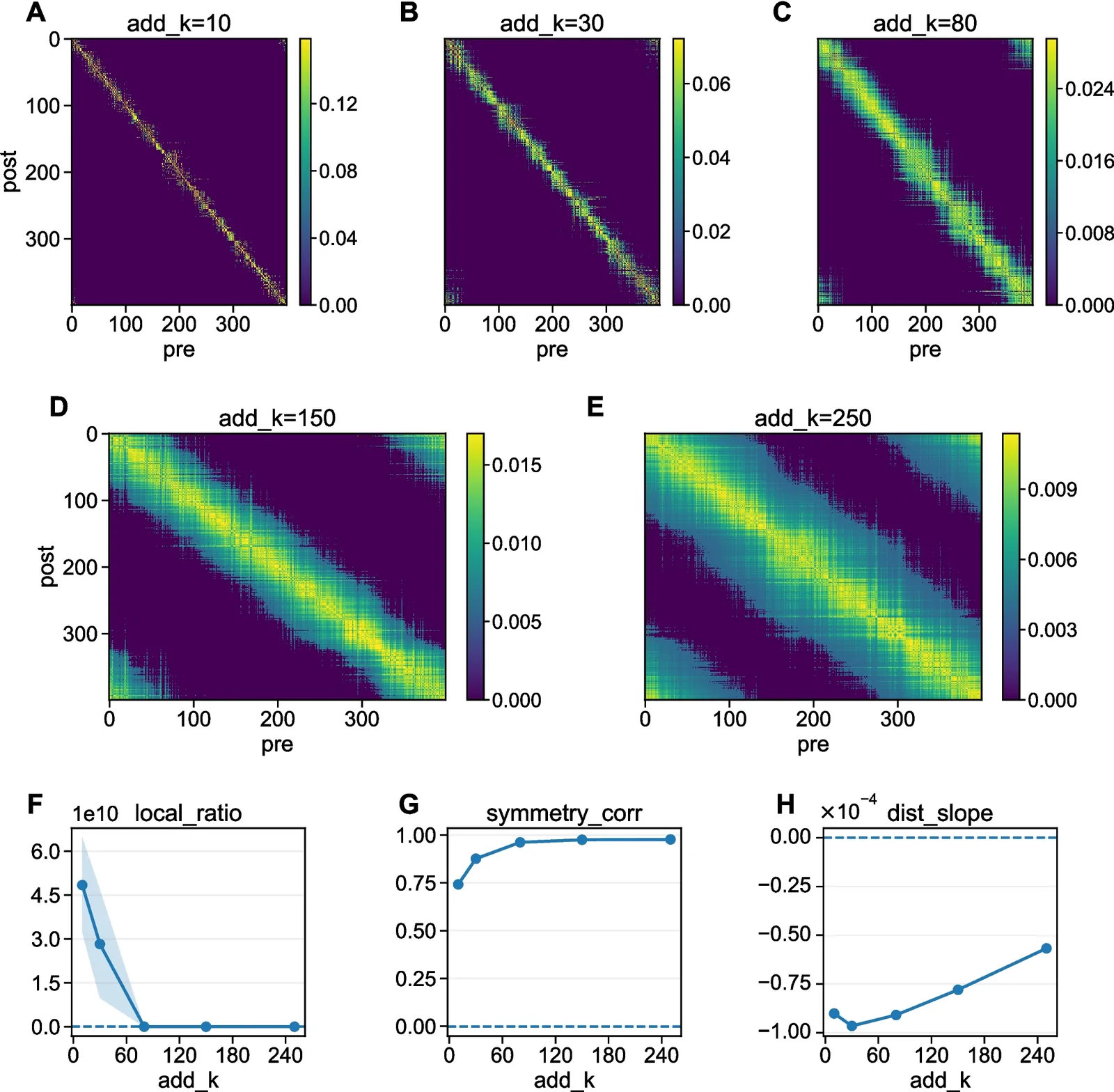
Structural-growth strength controls the width and sharpness of the learned local recurrent band **A–E**, representative phase-ordered recurrent weight matrices after learning with $k_{\textrm{add}}=10$, 30, 80, 150, and 250 new candidate connections per structural update; this parameter is labelled add_k in the figure, and colour denotes recurrent weight. **F–H**, corresponding locality, reciprocal-weight symmetry, and distance-dependent weight slope. Points and lines show the mean across *n=10* independently initialized simulation runs, shaded regions indicate mean ± 1 SD, and dashed horizontal lines denote the no-learning baseline. Increasing structural growth broadened and smoothed the local band while preserving its overall phase-organized structure

**Fig. 7:**
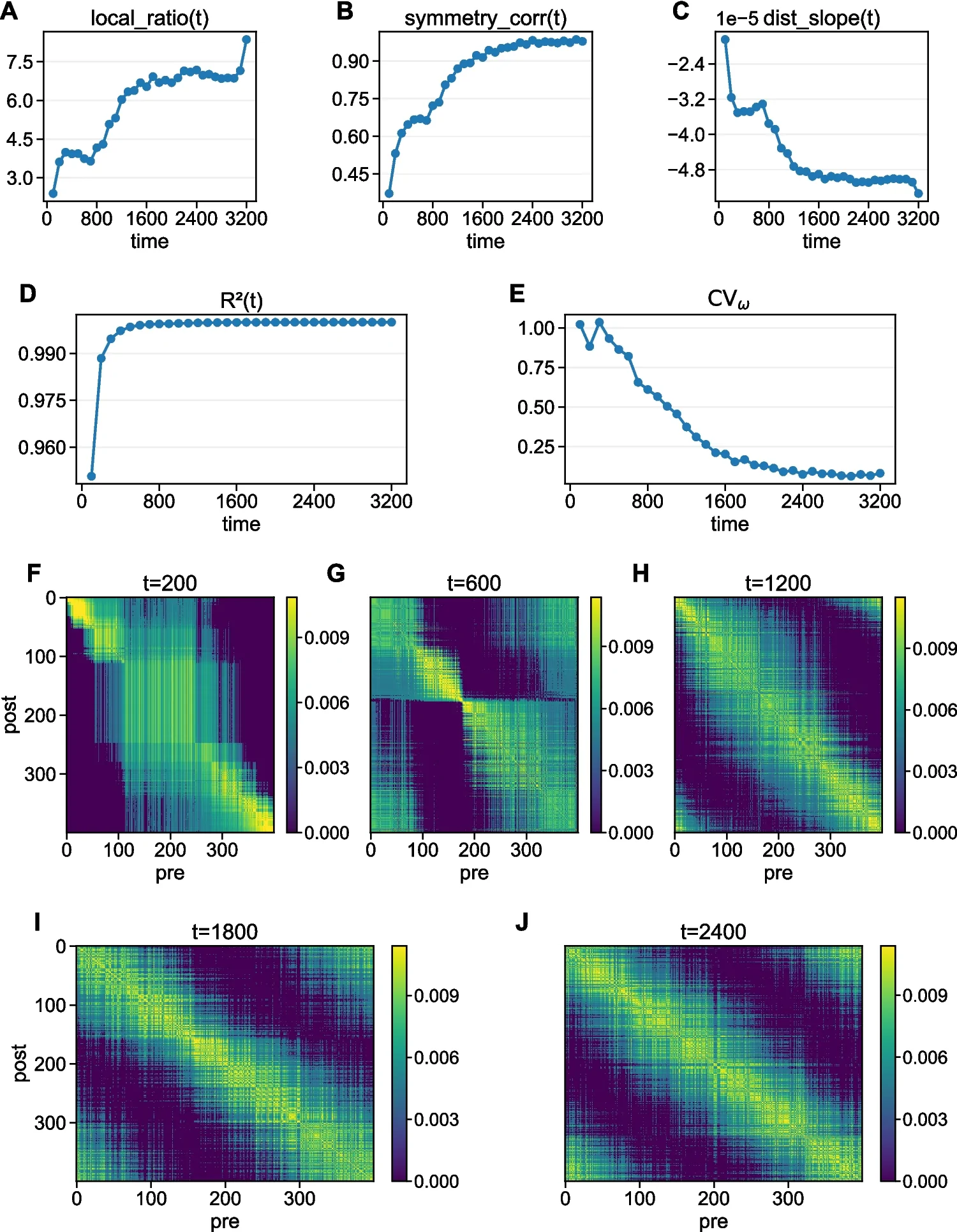
Local recurrent structure is progressively consolidated while travelling-wave dynamics remain stable **A–E**, temporal evolution of locality, reciprocal-weight symmetry, distance-dependent weight slope, phase-linearity $R^{2}$, and angular-speed unevenness $\textrm{CV}_{\omega }$ in a representative Ring-regime simulation with $k_{\textrm{add}}=250$ (labelled add_k in the figure). Each point summarizes the preceding analysis window; $R^{2}$ measures the linearity of unwrapped wave phase over time, and $\textrm{CV}_{\omega }$ is the coefficient of variation of the smoothed instantaneous phase velocity. **F–J**, phase-ordered recurrent weight matrices at five successive learning checkpoints; colour denotes recurrent weight. The initially coarse connectivity gradually developed into a continuous local band, while phase progression became highly regular and speed unevenness declined

Within the parameter regime that already supported waves, changing the strength of structural growth mainly altered the width and sharpness of the local band rather than its basic organization (Fig. 6). Weaker growth yielded a narrower and sharper band, whereas stronger growth produced a broader and smoother local structure. Figure 7 further shows that this structure emerged gradually. Early in learning, the matrix remained coarse and patchy; with time, a continuous enhancement zone developed near the main diagonal and matured into a stable local band. At the same time, the wave-quality measures remained stable, indicating that structure formation did not come at the expense of the wave scaffold. The travelling wave is therefore not a transient precursor, but a developmental template that is gradually consolidated into local recurrent structure.

The sensitivity analyses further distinguished parameters that were essential for structural consolidation from those that mainly modulated it (Appendix Figs. 11 and 12). Successful learning was robust across the tested STDP-like learning rates, depression factors, trace time constants, structural-decay rates, and most pruning thresholds. In contrast, insufficient growth capacity prevented formation of the target local structure: success was low when fewer than approximately 150 new edges were available per structural update or when updates were too infrequent. Increasing the number of new edges compensated for longer update intervals. A non-zero coactivation estimate and non-zero initial weight for newly added edges were also required, while removing the coactivation-pruning threshold preserved the travelling wave but prevented selective local structure from satisfying the learning criterion.

### Delayed inhibition stabilizes single-bump attractors

**Fig. 8:**
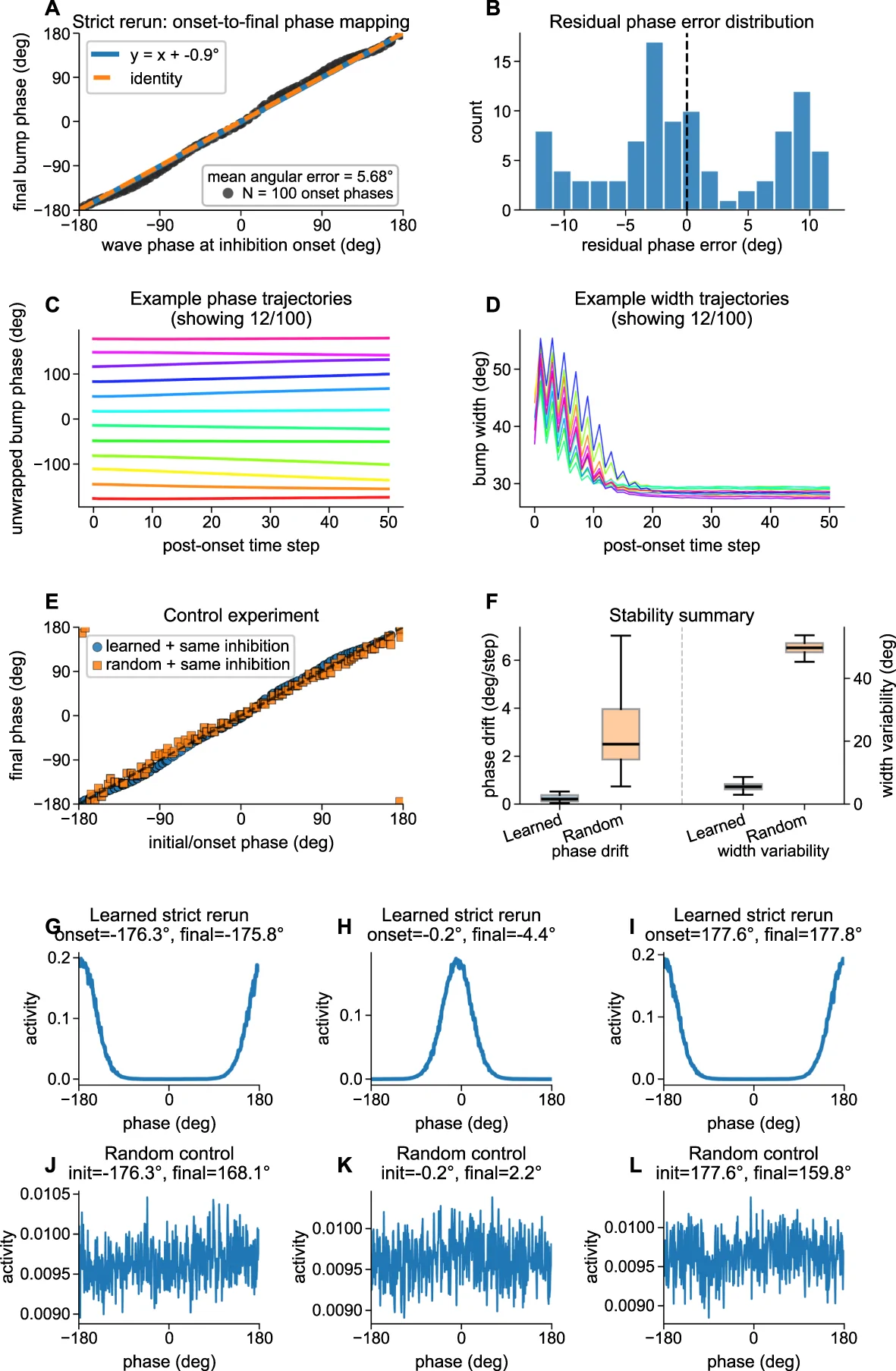
Delayed long-range inhibition converts the travelling-wave manifold into a phase-preserving family of stable bump states One hundred inhibition-onset times were selected to approximately cover the full travelling-wave phase cycle. These onset conditions are within-network replays of the same learned network rather than independently initialized network realizations. For each onset condition, the complete simulation was rerun, long-range inhibition was activated at the selected time, and the network was followed throughout the post-onset analysis interval. For each replay, the reported final phase was estimated as the circular mean of the bump phase over the stability-analysis window. **A**, final bump phase versus phase immediately before inhibition onset. Dark-grey circular markers show individual onset–final phase pairs. The solid blue line shows the circular fit $\theta _{\textrm{final}}=\theta _{\textrm{onset}}+\delta$, whereas the orange dashed line denotes the identity relation. **B**, distribution of wrapped residual phase errors from the circular fit. **C–D**, phase and bump-width trajectories for 12 representative onset conditions selected across the travelling-wave phase cycle. For clarity, only 12 of the 100 onset-state replays are displayed, whereas all 100 replays are retained for the quantitative analyses. Bump width is the circular standard deviation of the phase-ordered activity profile and is reported in degrees. **E**, onset-to-final mapping for the learned network and a random-connectivity control initialized from the same onset states and subjected to the same long-range inhibition; the black dashed line denotes the identity relation. **F**, distributions of phase drift and width variability. Phase drift is the mean absolute circular phase displacement per step, and width variability is the temporal standard deviation of bump width. Width variability is reported in degrees. Boxes show the interquartile range, centre lines indicate the median, and whiskers show the range of non-outlier values; outliers are not shown. **G–I**, final phase-ordered activity profiles from three representative learned-network reruns. **J–L**, corresponding random-network controls initialized from the same three onset states. The learned network preserved onset phase and formed stable localized bumps, whereas the random-connectivity control showed greater phase drift and width variability

We then asked whether long-range inhibition had to be present as an early template, or whether it could be recruited later. To test this, we performed strict replay analysis in the same post-learning network. Long-range inhibition was turned on at multiple time points spanning the full phase cycle of a stable travelling wave, and the phase at onset was compared with the final bump location. As shown in Fig. 8A–B, the final state preserved the phase information present at the moment inhibition was introduced: different initial phases converged to different final bump locations, and the phase-preservation error remained small. Figure 8C–D further show that different branches retained their original order while bump width rapidly contracted into a narrow range.

This stabilization was not generated by inhibition alone. When the same initial states and inhibitory term were used but the learned recurrent connectivity was replaced with random connectivity, the network failed to form comparably clear and stable local bumps and showed larger phase drift and bump-width fluctuation (Fig. 8E–F). Long-range inhibition therefore does not create the ring attractor from scratch. Rather, it compresses and stabilizes a local phase manifold that has already been established by learning.

Complementary noise-sensitivity analyses showed that the developmental sequence was more sensitive to Gaussian-noise statistics than the survival of an already formed bump (Appendix Fig. 13). Stage 3 bump stabilization was the most selective stage, whereas frozen networks retained high bump survival across most acute-noise conditions, although their positional stability remained noise dependent.

### DTN feedback learns direction-selective bump shifts

**Fig. 9:**
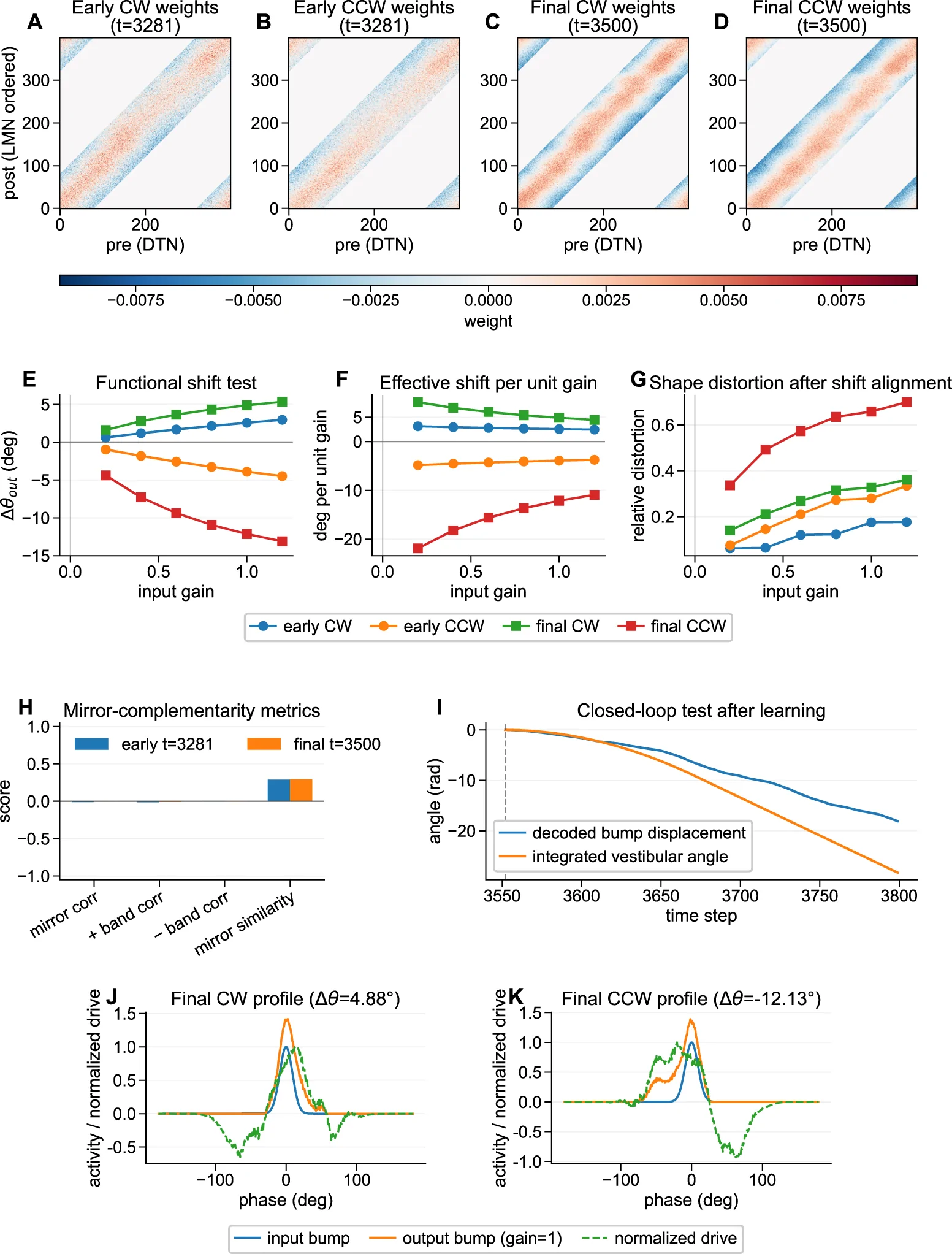
Learning produces direction-selective but imperfect DTN*→ *LMN shift operators **A–D**, clockwise (CW) and counterclockwise (CCW) DTN*→ *LMN weight matrices at early and final learning stages. Learning generated oppositely biased positive and negative sub-bands in the two pathways. **E**, angular displacement of a standardized activity bump produced by the learned operators across different input gains. **F**, effective shift per unit gain, calculated as the bump displacement divided by the applied gain. **G**, relative output-shape distortion after circularly aligning the shifted bump with the input profile. **H**, mirror complementarity between the CW and CCW operators, quantified by full-matrix correlation, positive- and negative-band correlations, and normalized mirror similarity. **I**, closed-loop bump displacement compared with the cumulative angle obtained by integrating the imposed vestibular angular-velocity signal. **J–K**, representative final CW and CCW responses to a standardized bump at unit gain; the blue and orange solid curves show the input and output bump profiles, respectively, and the green dashed curve shows the normalized operator drive. The learned pathways shifted the bump in opposite directions and supported closed-loop updating, but retained directional imbalance, shape distortion, and gain mismatch

We next asked whether the DTN*→ *LMN pathway could acquire direction-selective translation properties from wave-related temporal statistics alone. Figure 9A–D show that during learning the DTN*→ *LMN matrices evolved from unstructured noise into phase-biased feedback kernels with recognizable positive and negative sub-bands. To test function directly, we applied DTN feedback of different strengths to a standardized bump and measured the resulting peak displacement. Figure 9E–F show that the clockwise pathway consistently shifted the bump in the positive direction, whereas the counterclockwise pathway shifted it in the opposite direction. The displacement produced by late-learning feedback was also larger than that produced early in learning, indicating progressive strengthening of direction-selective shift function.

At the same time, the learned feedback was not an ideal translation operator. As input strength increased, the output bump showed increasing shape distortion even after realignment (Fig. 9G), and the two directions remained unequal in translation efficiency. When the learned feedback was inserted back into the closed-loop network, the bump moved in the correct direction but cumulative displacement gradually deviated from the theoretical integral (Fig. 9I). Thus, the DTN pathway learned usable direction-selective shift feedback, but not an ideal error-free translation rule.

### Visual calibration improves gain but leaves residual drift

**Fig. 10:**
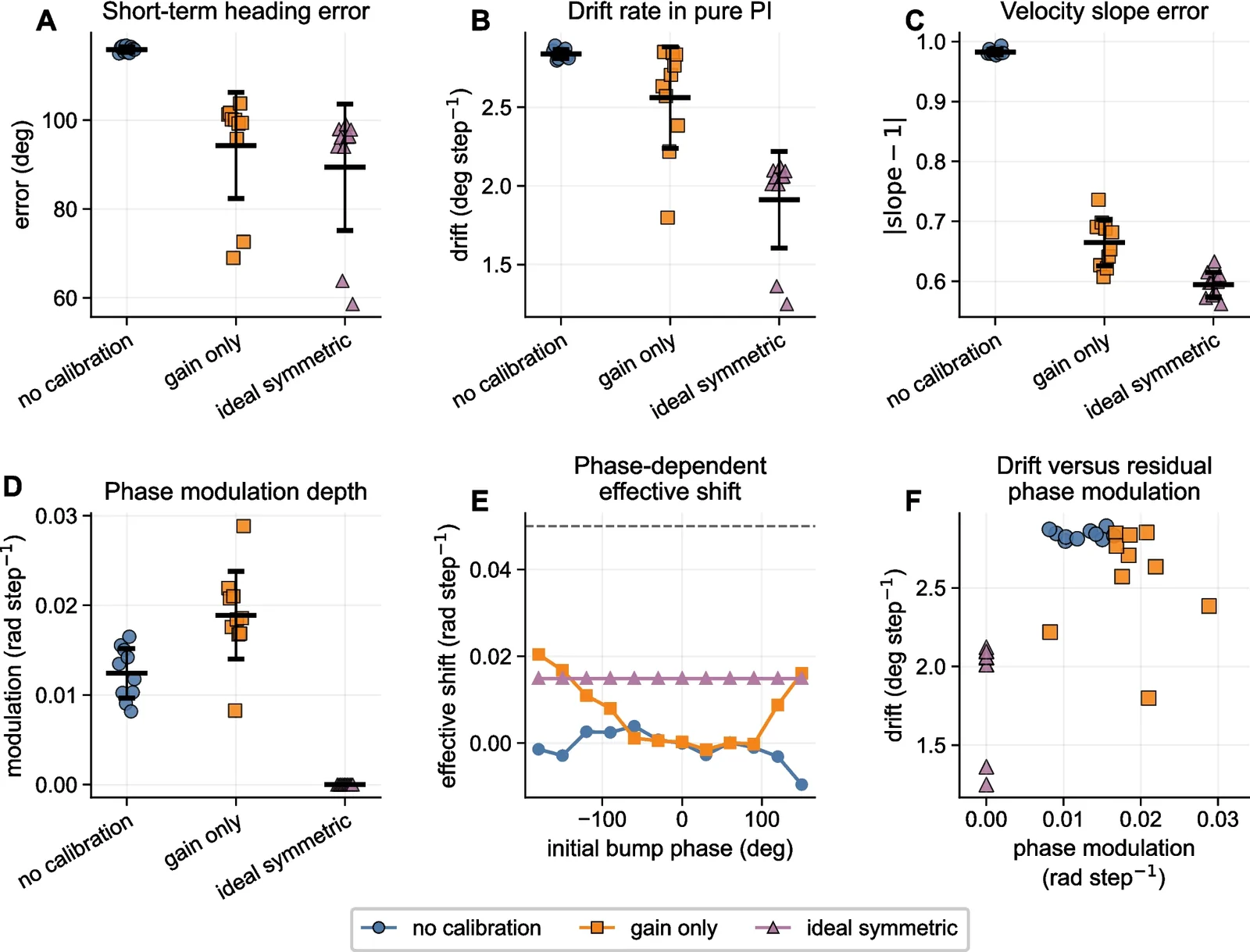
**Gain calibration improves path-integration performance but does not eliminate phase-dependent residual error** Three conditions were compared: *no calibration*, which retained the pre-visual DTN-to-LMN operator and its initial expression gain; *gain only*, which retained the same operator while optimizing the DTN gain; and the *ideal-symmetric condition*, in which the recurrent LMN connectivity and the pre-visual DTN-to-LMN operator were symmetrized before separate gain optimization. **A–D**, coloured points represent *n=10* independently initialized simulation runs; black horizontal bars show the across-run mean, and vertical error bars indicate mean ± 1 SD. **A**, short-term heading-error RMSE during the initial portion of a constant-angular-velocity probe. **B**, absolute long-term drift rate during the later portion of the same probe, PI denotes path integration.. **C**, absolute velocity-slope error, *|b-1|*, where *b* was obtained from a zero-intercept regression of decoded against imposed angular velocity during a sinusoidally varying angular-velocity probe. **D**, phase-modulation depth, defined as the range of effective shift speeds across initial bump phases. **E**, phase-dependent effective shift speed for a representative run; the dashed line denotes the imposed angular velocity. **F**, relationship between phase-modulation depth and pure path-integration drift across runs. All performance probes were conducted after visual-cue removal under purely vestibular drive. Gain-only calibration reduced short-term error and average drift but retained greater phase dependence than the *ideal-symmetric condition*.

Finally, we asked whether late visual calibration could turn the developmentally formed network into an ideal drift-free integrator. Figure 10 compares the uncalibrated network, the gain-only calibrated network, and an idealized control. Figure 10A–C show that visual calibration primarily corrected a coarse velocity mismatch: after adjusting only the overall gain, short-term directional error was substantially reduced and the average angular-velocity match improved markedly. However, the gain-only condition remained systematically worse than the idealized control, showing that scalar calibration alone was insufficient to restore ideal integration.

Figure 10D–E further show that long-term drift persisted because bump motion remained phase dependent: even after average gain was corrected, some phases advanced faster and others more slowly. This indicates that the developmentally formed network retained local structural inhomogeneities, so that its translation rule was not perfectly equivariant. In the present setting, visual calibration mainly repaired the macroscopic gain mismatch, thereby substantially improving short-timescale performance and partially reducing drift, but it did not eliminate the microscopic phase dependence. The developmental HD circuit is therefore better described as an approximate integrator than as an ideal one.

Additional sensitivity analyses across visual amplitude, width, temporal profile, and presentation duration showed that calibration was not restricted to a single visual-input profile, although long-horizon performance depended on the spatial and temporal structure of the available visual signal (Appendix Fig. 14).

## Discussion

The present study addresses a central gap in the HD literature: although the function of mature HD circuits has been characterized in considerable detail, existing models do not explain how internal coherence can emerge before stable sensory anchoring. Classical ring-attractor models usually begin from circuits in which local excitation, long-range inhibition, and direction-selective shift feedback are already in place (Angelaki & Laurens, 2020; Song & Wang, 2005). Here we propose a staged developmental account in which these components arise progressively from spontaneous dynamics and local plasticity.

In the model, a randomly connected LMN network first generates repeatable travelling waves. STDP-like and structural plasticity then consolidate the temporal statistics of these waves into local recurrent structure. Long-range inhibition, recruited later, converts this propagating manifold into a family of stable single-bump attractor states. Visual input subsequently acts mainly as a calibration signal for overall integration gain. In this sense, the model supports a dynamics-first, structure-later, calibration-last developmental scenario. The central point is not that the mature HD circuit must be built from an initially perfect attractor, but that an internally coherent manifold may emerge first and only later become stabilized and aligned to the external world.

This framework provides a natural account of a key developmental observation: before eye opening, relative HD coherence is already present, whereas absolute directional tuning remains unstable (Bjerknes et al., 2015; Tan et al., 2015). In the model, these two features arise from different stages of development. Internal population geometry is established first, initially as travelling-wave dynamics and later as a stable attractor. Alignment to external reference, by contrast, depends on later sensory calibration. The model therefore suggests that the formation of internal representational structure and the anchoring of that structure to the outside world are separable developmental processes.

A further implication concerns the role of long-range inhibition. In many mature ring-attractor models, inhibition is part of the initial architecture. Here, by contrast, long-range inhibition acts on a substrate that has already been partially organized by spontaneous activity and learning. Its function is not to create the ring manifold de novo, but to compress and stabilize an existing local phase manifold (Amari, 1977; Page et al., 2018). This suggests that the mature attractor may be assembled in stages: first as an ordered propagating state, then as local recurrent structure, and finally as a stable continuous attractor.

The model also provides an account of why real HD circuits may remain non-ideal. Because the learned recurrent and DTN feedback structures retain residual heterogeneity, the resulting integrator is not perfectly equivariant. Visual calibration substantially improves short-timescale accuracy by correcting the dominant gain mismatch, but it does not remove phase-dependent error completely. The developmental HD circuit is therefore better understood as a good-enough integrator than as a mathematically ideal one (Tocker et al., 2017; Vafidis et al., 2022). In this view, long-term drift is not simply a defect to be ignored, but a natural consequence of developmental self-organization.

The present study has several limitations. The model is rate-based and does not explicitly represent spiking dynamics or detailed synaptic mechanisms. Visual input is simplified as a calibration signal rather than implemented through a full sensory pathway. In addition, phase ordering is used here as an external analysis coordinate rather than as an explicitly embedded biological variable. Future work could extend the framework by incorporating more detailed neural dynamics, multimodal calibration, and downstream circuits such as ADN. It will also be important to test experimentally whether early LMN–DTN dynamics, developing DTN*→ *LMN feedback, and later visual calibration are dissociable stages of HD circuit formation. The present study does not examine networks with an explicit two- or three-dimensional spatial embedding; determining whether the same developmental mechanism generalizes to such settings remains an important direction for future work. Overall, the model provides a mechanistic framework linking early internal organization to the later emergence of mature HD computation.

## Conclusion

We constructed a developmental model of the LMN–DTN head direction circuit in which a functional HD ring attractor emerges through staged self-organization without preconfigured topology or early external supervision. In the model, travelling waves in a random LMN network provide the temporal scaffold; local plasticity consolidates this scaffold into recurrent structure; delayed long-range inhibition stabilizes a family of single-bump attractor states; and late visual input calibrates overall integration gain. The resulting circuit supports internal coherence and direction updating, but retains long-term drift and phase-dependent error. The model therefore provides a mechanistic account of how early internal organization can precede stable sensory anchoring, and suggests that key features of classical ring-attractor models may arise through development rather than being present from the outset.

## Figures and Tables

**Fig. 11:**
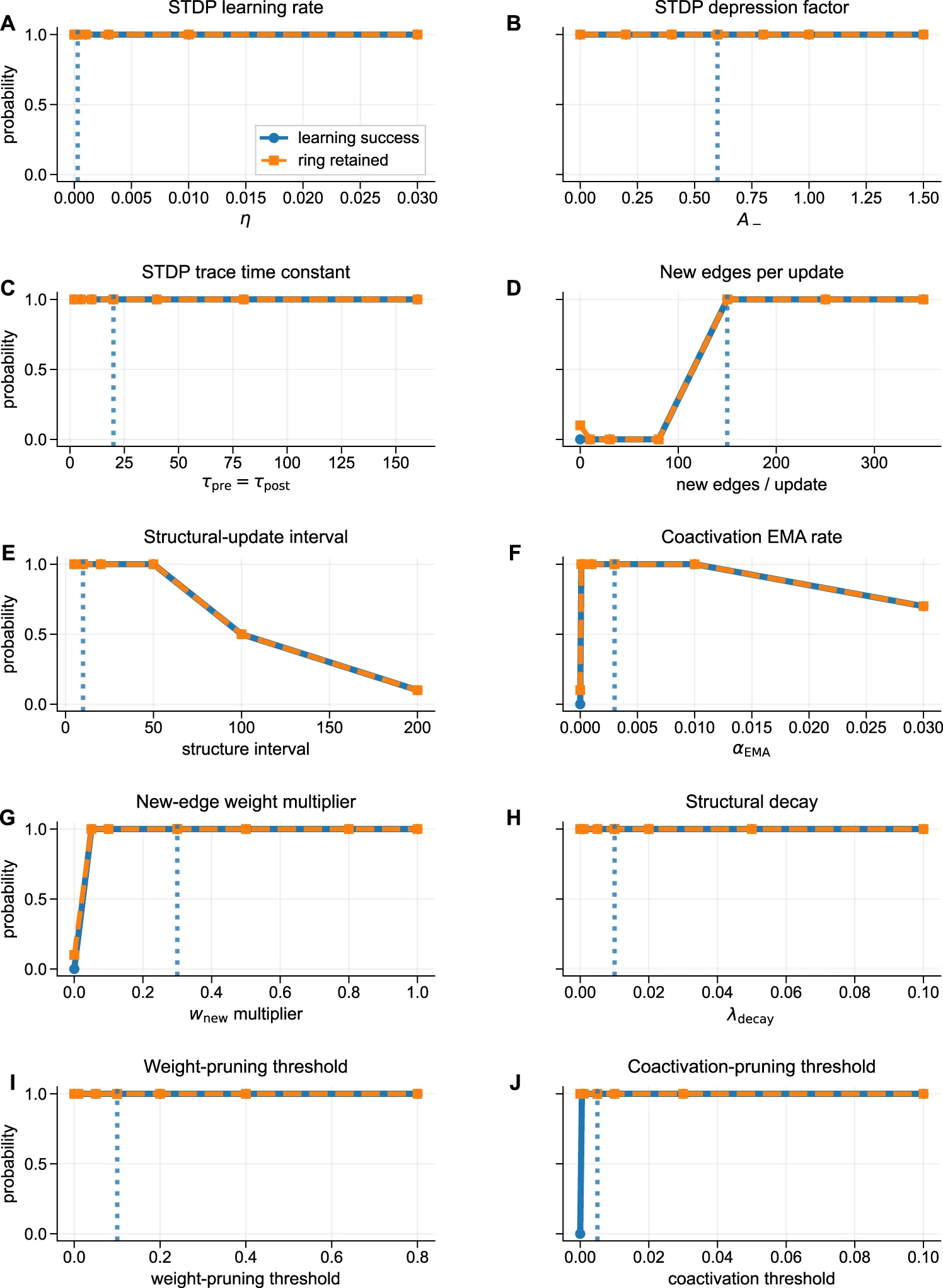
One-parameter sensitivity of travelling-wave retention and Stage 2a recurrent and structural learning **A–J**, each parameter was varied individually while all other parameters were fixed at their baseline values, indicated by the dotted vertical lines. The two curves show the fractions of *n=10* independent runs satisfying the ring-retention and learning-success criteria, respectively. A run retained the travelling wave when $R^{2}\ge 0.9$, $\textrm{CV}_{\omega }\le 0.2$, $\textrm{CV}_{r}\le 0.30$, and at least two propagation cycles were present. Learning success required ring retention together with $\mathrm {local\_ratio}>1.05$, $\mathrm {symmetry\_corr}>0.25$, $\mathrm {dist\_slope}<0$, mean phase-tuning strength *>0.25*, and median tuning width $<160^{\circ }$. Thus, a probability of 1 indicates that all 10 runs passed the corresponding criterion, whereas 0 indicates that none did. Structural learning was most sensitive to the number and initial strength of newly added edges, the structural-update interval, and the coactivation estimate, but was comparatively robust to broad variation in the remaining plasticity parameters

**Fig. 12:**
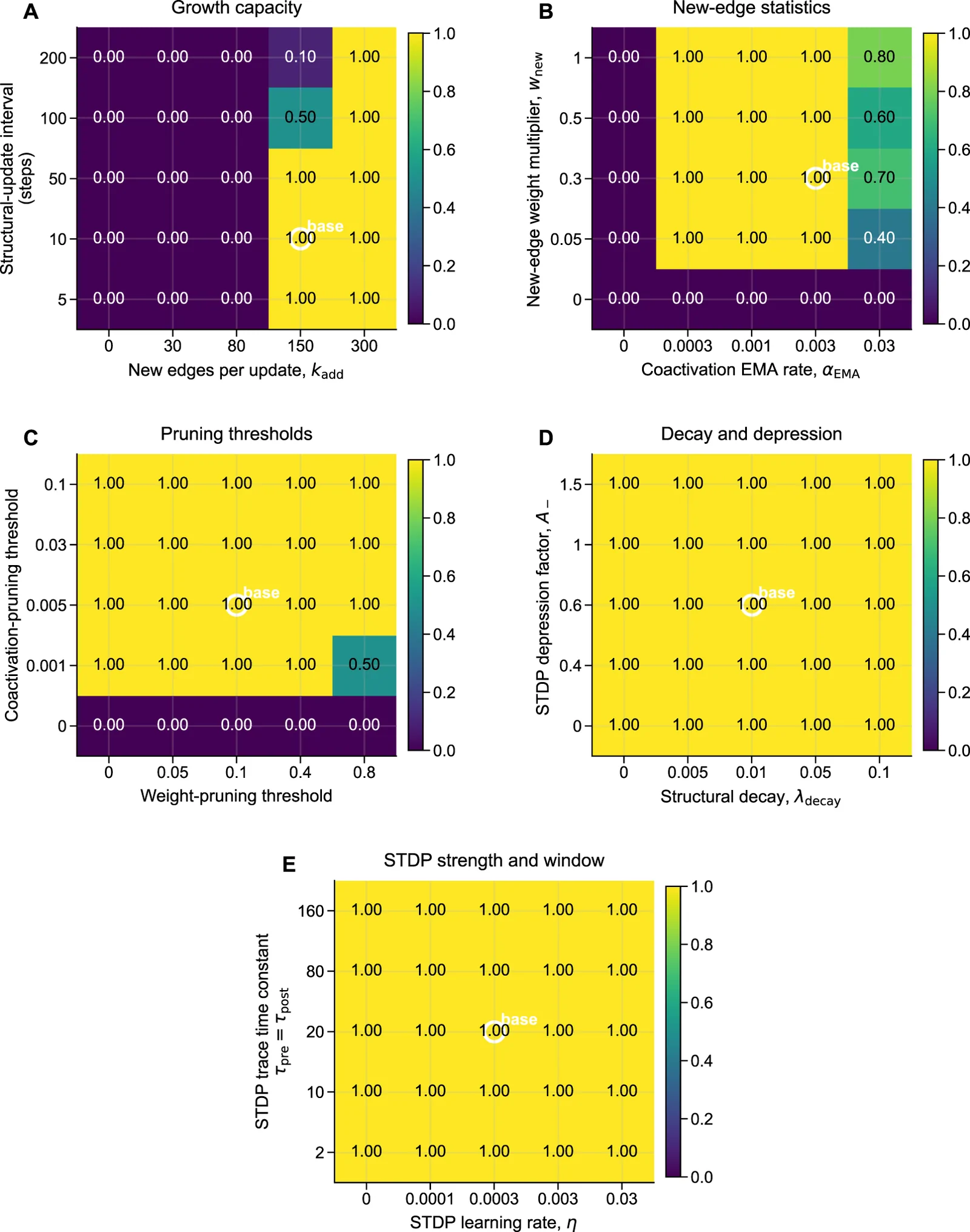
Pairwise parameter sensitivity of Stage 2a structural-learning success **A–E**, paired scans of growth capacity, new-edge statistics, pruning thresholds, structural decay and depression, and STDP-like strength and trace duration. Colour and the value printed in each cell indicate the learning-success probability, calculated as the fraction of *n=10* independent runs that passed the complete Stage 2a criterion; these values therefore do not represent $P(\textit{ring})$ alone. For example, 1.00 indicates success in all 10 runs and 0.50 indicates success in 5 of 10 runs. A successful run had to retain a stable travelling wave ($R^{2}\ge 0.9$, $\textrm{CV}_{\omega }\le 0.2$, $\textrm{CV}_{r}\le 0.30$, and at least two cycles) and develop the target recurrent structure ($\mathrm {local\_ratio}>1.05$, $\mathrm {symmetry\_corr}>0.25$, $\mathrm {dist\_slope}<0$, mean phase-tuning strength *>0.25*, and median tuning width $<160^{\circ }$). The white circled marker labelled “B” in each panel denotes the baseline parameter combination. The results identify growth capacity and new-edge formation as the principal constraints, whereas broad regions of the STDP-like, decay, and pruning parameter spaces retained successful learning.

**Fig. 13:**
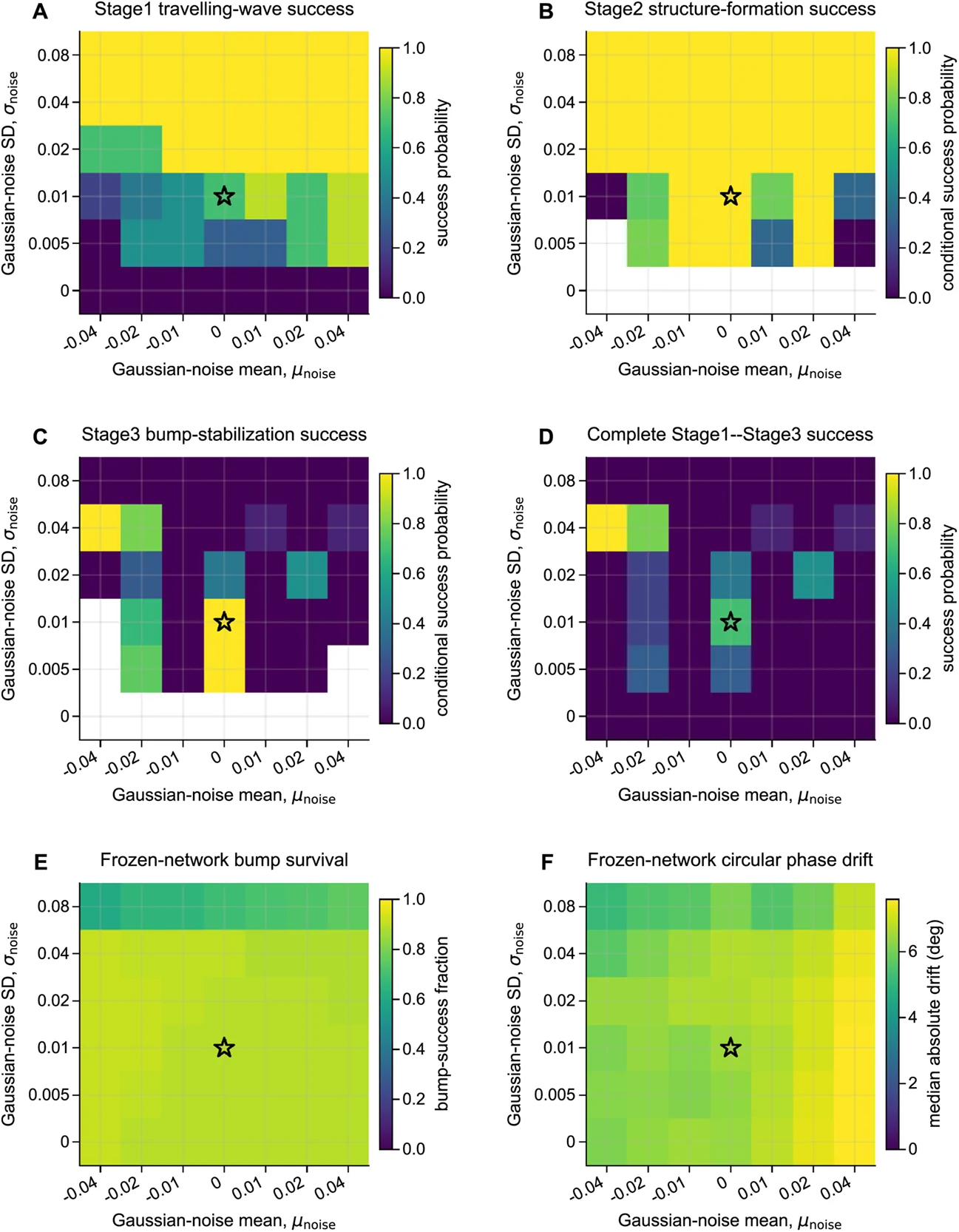
Gaussian-noise mean and standard deviation affect successive developmental stages differently Gaussian noise was drawn as $\boldsymbol{\xi }^{\textrm{noise}}_{t} \sim \mathcal {N}(\mu _{\textrm{noise}}, \sigma _{\textrm{noise}}^{2})$. Ten independently initialized networks were tested for each combination of noise mean and standard deviation; stars mark the baseline setting $(\mu _{\textrm{noise}},\sigma _{\textrm{noise}})=(0,0.01)$. **A**, Stage 1 travelling-wave success during the pre-learning interval. **B**, Stage 2a recurrent-structure formation success during the Stage 2 learning interval, conditional on successful Stage 1 development. **C**, Stage 3 bump-stabilization success, evaluated after Stage 2 and conditional on successful Stage 2a recurrent-structure formation. Twelve onset states sampled late in Stage 2 were replayed for each eligible network, and Stage 3 was considered successful when at least 80% formed stable bumps. **D**, probability that the complete developmental sequence across Stages 1–3 succeeded. **E–F**, acute-noise tests in frozen networks developed under the baseline noise condition: bump-survival fraction and median absolute circular phase drift, respectively. White cells in the conditional maps indicate parameter combinations for which no network passed the preceding developmental stage and the subsequent stage was therefore not evaluated; these cells do not represent a success probability of zero

**Fig. 14:**
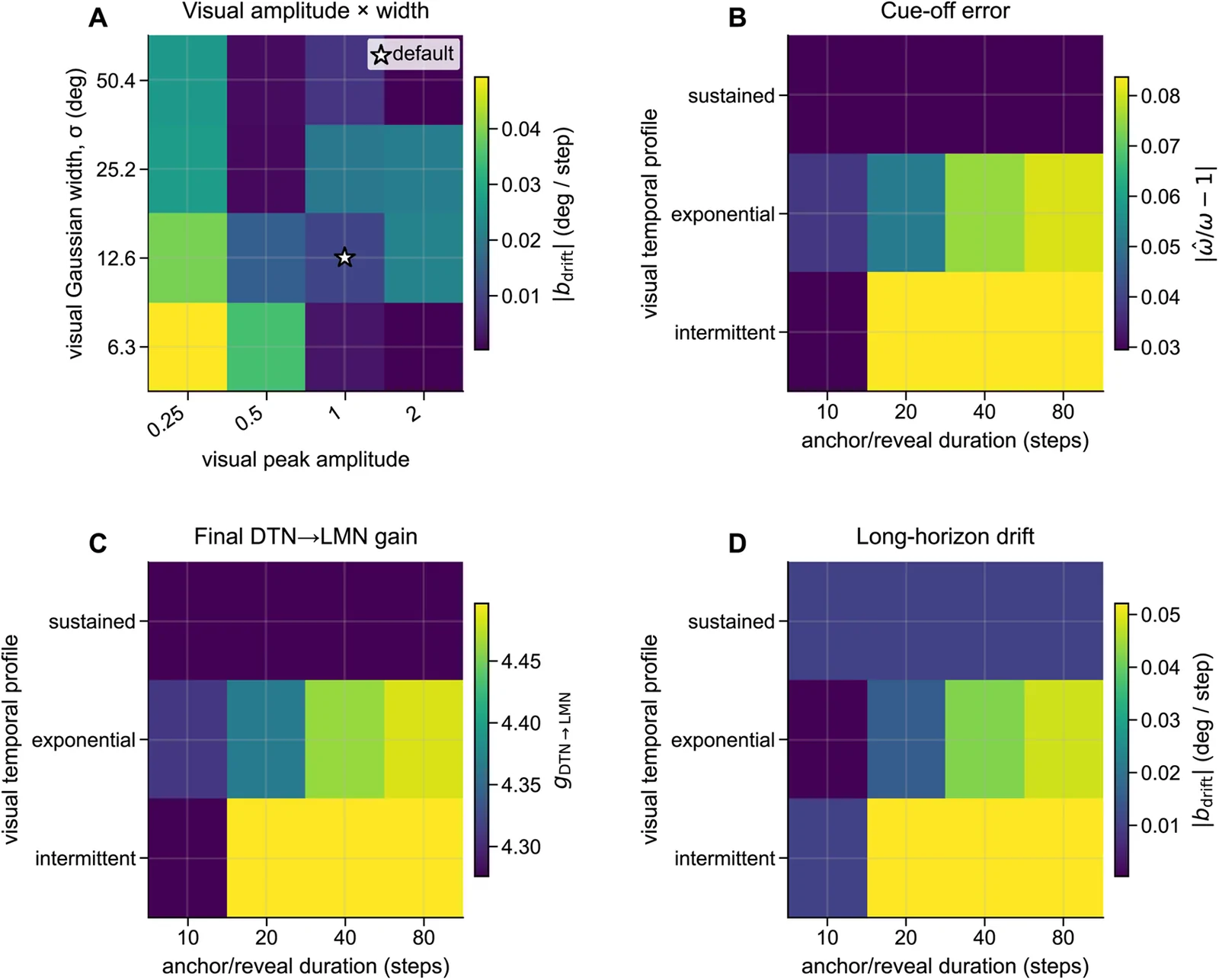
Path-integration performance depends on the spatial and temporal form of the visual calibration signal **A**, absolute long-horizon cue-off drift rate, $|b_{\textrm{drift}}|$, across visual peak amplitudes $A_{\textrm{VIS}}=\{0.25,0.5,1,2\}$ and Gaussian widths $\sigma _{\textrm{VIS}}=\{6.3^{\circ },12.6^{\circ },25.2^{\circ },50.4^{\circ }\}$; the filled star marks the selected reference setting used for the temporal-profile analyses, whereas the outlined star labelled “default” marks the original default setting. **B–D**, effects of three visual temporal profiles across a range of visual-presentation durations. The *sustained* input remained constant throughout each visual-presentation window; the *exponential* input followed $s(u)=\exp (-u/\tau _{\textrm{VIS}})$, where $\tau _{\textrm{VIS}}$ is the visual decay time constant; and the *intermittent* input alternated between on and off epochs. All three temporal profiles used the same peak visual-input amplitude, but their time-integrated visual drive was not matched; consequently, they provided different cumulative visual input over the same presentation duration. Visual input was absent during the subsequent cue-off test. **B**, cue-off velocity-gain error, $|\hat{\omega }/\omega -1|$. **C**, final calibrated scalar DTN gain $g_{\textrm{DTN}}$. **D**, absolute long-horizon cue-off drift rate, defined as the fitted slope of heading error over time. Each parameter combination underwent 60 calibration episodes within the frozen learned network. Long-horizon drift was evaluated without visual input during the cue-off constant-angular-velocity probe and averaged across three independent noise realizations. These repeated calibration episodes and cue-off noise realizations probe within-network calibration robustness and are not independent network initializations

## Data Availability

The simulation data and code generated and analyzed during this study are available from the author upon reasonable request.

## Appendix: Sensitivity and robustness analyses

This Appendix contains the parameter-sensitivity and robustness analyses referred to in the main text. Unless otherwise stated, model definitions, success criteria, reference parameter values, and statistical conventions are identical to those described in the main manuscript.

### Stage 2a recurrent and structural learning sensitivity

Appendix Figs. 11 and 12 provide the one-parameter and pairwise sensitivity analyses underlying the main-text summary of Stage 2a recurrent and structural learning. These analyses distinguish parameters that constrain successful structural consolidation from those that mainly modulate the learned recurrent organization.

### Noise sensitivity across developmental stages

We examined how the mean and standard deviation of Gaussian noise influenced the sequential developmental process across Stages 1–3 (Appendix Fig. 13). The analysis followed the actual temporal dependency of the model: travelling-wave formation was assessed during Stage 1 (steps 0–800), Stage 2a recurrent-structure formation was evaluated within the Stage 2 learning interval (steps 800–3200) only in networks that had passed Stage 1, and bump stabilization was subsequently tested by activating long-range inhibition from states sampled late in Stage 2, between steps 3000 and 3200. Stage 1 required non-zero noise variability and became increasingly reliable over a broad range of noise means as the noise standard deviation increased. Conditional on successful wave formation, Stage 2a recurrent-structure formation was comparatively robust. Stage 3 bump stabilization was more selective and non-monotonic, producing a narrower successful region and therefore acting as the principal bottleneck for complete developmental success. In contrast, when already developed baseline networks were frozen, bump survival remained high across most acute-noise conditions, although circular phase drift varied with both the noise mean and standard deviation. Thus, noise affects the establishment of the developmental sequence more strongly than the mere survival of an already formed bump, while still influencing its positional stability.

### Sensitivity to the spatial and temporal form of visual calibration

To determine whether calibration depended on the precise form of the visual input, we additionally varied the peak amplitude and Gaussian spatial width of the visual signal and compared sustained, exponentially decaying, and intermittent temporal profiles over different presentation durations (Appendix Fig. 14). Long-horizon drift depended on the interaction between visual amplitude and width. Sustained visual input was comparatively robust across presentation durations, whereas exponentially decaying and intermittent inputs produced stronger duration-dependent changes in the learned gain, cue-off velocity error, and subsequent drift. Thus, calibration was not restricted to a single visual-input profile, but its effectiveness depended on the spatial and temporal structure of the available visual evidence.
